# Molecular apocrine differentiation is a common feature of breast cancer in patients with germline *PTEN *mutations

**DOI:** 10.1186/bcr2626

**Published:** 2010-08-16

**Authors:** Guillaume Banneau, Mickaël Guedj, Gaëtan MacGrogan, Isabelle de Mascarel, Valerie Velasco, Renaud Schiappa, Valerie Bonadona, Albert David, Catherine Dugast, Brigitte Gilbert-Dussardier, Olivier Ingster, Pierre Vabres, Frederic Caux, Aurelien de Reynies, Richard Iggo, Nicolas Sevenet, Françoise Bonnet, Michel Longy

**Affiliations:** 1 INSERM U916, Université de Bordeaux, Institut Bergonié, 229 cours de l'Argonne, 33000, Bordeaux, France; 2 Tumor Identity Card program (CIT3), Ligue Nationale Contre le Cancer, 12 rue Corvisart, 75013 Paris, France; 3 Pathology Department, Institut Bergonié, 229 cours de l'Argonne, 33000, Bordeaux, France; 4 Cancer Genetics Unit, Centre Léon Bérard, 28 rue Laennec, 69008 Lyon, France; 5 Medical Genetics Unit, CHU de Nantes, 5 allée de l'Île Gloriette, 44000 Nantes, France; 6 Cancer Genetics Unit, Centre Eugène Marquis, avenue de la Bataille Flandres-Dunkerque, 35000 Rennes, France; 7 Medical Genetics Unit, CHU de Poitiers, 2 rue Milétrie, 86000 Poitiers, France; 8 Medical Genetics Unit, CHU d'Angers, rue Larrey, 49100 Angers, France; 9 Dermatology Department, CHU de Dijon, 2 boulevard du Maréchal de Lattre de Tassigny, 21000 Dijon, France; 10 Dermatology Department, Hôpital Avicenne, 125 rue Stalingrad, 93000 Bobigny, France; 11 Cancer Genetics Unit, Institut Bergonié, 229 cours de l'Argonne, 33000 Bordeaux, France

## Abstract

**Introduction:**

Breast carcinoma is the main malignant tumor occurring in patients with Cowden disease, a cancer-prone syndrome caused by germline mutation of the tumor suppressor gene *PTEN *characterized by the occurrence throughout life of hyperplastic, hamartomatous and malignant growths affecting various organs. The absence of known histological features for breast cancer arising in a *PTEN*-mutant background prompted us to explore them for potential new markers.

**Methods:**

We first performed a microarray study of three tumors from patients with Cowden disease in the context of a transcriptomic study of 74 familial breast cancers. A subsequent histological and immunohistochemical study including 12 additional cases of Cowden disease breast carcinomas was performed to confirm the microarray data.

**Results:**

Unsupervised clustering of the 74 familial tumors followed the intrinsic gene classification of breast cancer except for a group of five tumors that included the three Cowden tumors. The gene expression profile of the Cowden tumors shows considerable overlap with that of a breast cancer subgroup known as molecular apocrine breast carcinoma, which is suspected to have increased androgenic signaling and shows frequent *ERBB2 *amplification in sporadic tumors. The histological and immunohistochemical study showed that several cases had apocrine histological features and expressed GGT1, which is a potential new marker for apocrine breast carcinoma.

**Conclusions:**

These data suggest that activation of the ERBB2-PI3K-AKT pathway by loss of PTEN at early stages of tumorigenesis promotes the formation of breast tumors with apocrine features.

## Introduction

The classification of breast cancer was recently enriched by the addition of gene expression microarray data for the main histopathological tumor types [1]. The most widely used transcriptomic classification, the Intrinsic Gene Set or Stanford classification, divides breast cancer into luminal A, luminal B, basal-like, normal-like and HER2 classes [2-4]. It is based on studies of sporadic tumors, and reflects mainly tumor cell type and HER2 status. Few studies have looked at the gene expression profile of breast cancers arising in patients with a familial predisposition to cancer. In those studies, the tumors arising in patients with germline BRCA1 mutations frequently had a basal-like phenotype, whereas *BRCA2*-related tumors had no particular type or a luminal B type [5]. Little is known about the transcriptomic profile of breast cancers caused by germline mutations in genes other than *BRCA1 *and *BRCA2*. Cowden disease (MIM 158350) is a cancer predisposition syndrome caused by germline mutation of the tumor suppressor gene *PTEN *[6,7]. In addition to hamartomas and hyperplasias affecting multiple organs, patients develop breast cancer with a cumulative risk > 50% at age 70 [8]. To investigate the pathogenesis of breast cancer in Cowden disease, we have analyzed the gene expression profile of three breast carcinomas from Cowden disease patients with known germline *PTEN *mutations.

## Materials and methods

### Patients and samples

To allow us to identify distinctive features of PTEN-related tumors, the analysis was performed in the context of a panel of 74 tumors from patients with a familial clustering of breast cancer, including 7 with *BRCA1 *and 5 with *BRCA2 *mutations. All of the samples were taken from the Bergonié Cancer Institute tumor bank. The patients belong to families with either i) at least three cancer affected first degree relatives including at least two with breast cancer; or ii) two first degree relatives with breast cancer at a young age of onset (mean age up to 50 years). Only one tumor sample per patient was tested. In most cases only one tumor was available per family, but in one family, three samples were available, and in seven other families, including one Cowden family, two samples were available. Major demographic, clinical and pathological features are listed in Table S1 in Additional file 1. All patients agreed to the use of their samples for research purposes, in compliance with the French law on tumor banks (law n° 2004-800). The *PTEN *mutation search was made after signed informed consent in the context of a medical genetic diagnosis of suspected Cowden disease, in compliance with the French law on genetic testing (law n° 94-654).

### Gene expression analysis

After assessment of tumor cellularity in each sample on haematoxylin-eosin stained frozen sections, Rneasy Mini Kits (Qiagen S.A., Courtaboeuf, France) were used to extract total RNA from samples ground to powder while frozen. RNA quality was assessed using the Agilent 2100 Bioanalyzer (Agilent Technologies France, Massy, France). Gene-expression analyses were performed by the IGBMC and Génopole Alsace-Lorraine Affymetrix service using Affymetrix U133 Plus 2.0 genechip microarrays(Affymetrix UK Ltd, High Wycombe, UK). The transcriptomics data are available in ArrayExpress database [9] - (accession number: [E-TABM-854]) or in the CIT database [10].

### Mutation analysis

DNA was purified from leucocytes and tumors by phenol-chloroform extraction. PTEN point mutations were identified by denaturing gradient gel electrophoresis (DGGE) screening followed by sequencing of the variants on an ABI DNA sequencing machine [11]. Large rearrangements were screened for by quantitative multiplex PCR [12].

### Immunohistochemistry

Immunohistochemical detection was performed with the REAL EnVision Detection System (Dako^®^, Trappes, France). Tissue microarraying was performed with a tissue arrayer (MTA Booster 01, Alphelys, Plaisir, France). For each sample, four 0.6 mm core sections of tissue were extracted from paraffin-embedded tissues. The following antibodies were used: anti-GGT1 (Sigma-Aldrich^® ^St. Louis, MO, USA, clone 1F9); anti-PTEN (Cascade Biosciences™ Winchester, MA, USA, clone 6H2.1); anti-Estrogen receptor (Dako^® ^Trappes, France, clone 1D5); anti-Progesterone receptor (Dako^®^, Trappes, France, clone PgR636); anti-HER2 (Dako^®^, Trappes, France, cloneAO485); anti-Androgen Receptor (Dako^® ^Trappes, France, clone 1D5); anti-GCDFP15 (Signet™, Dedham, MA, USA, clone D6); anti-EGFR (Ventana^®^, Illkrich, France, clone 3C6). Two parameters were evaluated for each antibody: (i) the percentage of tumoral cells showing a positive signal and (ii) the intensity of that signal classed as low (1), moderate (2) or high (3). ERBB2 expression was evaluated according to the Herceptest scoring system. For ER, PR, AR, PTEN, GCDFP15, GGT1 and EGFR, the following scoring was used: score 0, no tumor cells with any positivity; score1, 1 to 10% of tumors cells showing a positive signal; score 2, 11 to 100% of tumor cells showing a positive signal, whatever associated intensity of staining.

### Array CGH analyses

Array CGH was performed on human Integrachip V7 slides (Integragen SA, Evry, France, [13]). IntegraChip V7 is composed of 5878 BAC clones with a median of 0.5 Mb between clones. BAC clones are spotted in quadruplicate. A pool of 19 normal DNAs was used as reference DNA. DNA was labelled by random priming with cyanine 5 for reference DNA (Cy5) and cyanine 3 for tumor DNA (Cy3). Hybridizations were performed according to the manufacturer's recommendations. Slides were scanned with an Axon 4000B scanner (Axon Instruments Inc., Union City, CA, USA) and acquired images were analyzed with GenePix Pro 5.1 image analysis software to perform segmentation and to determine the mean intensities for the Cy3 and Cy5 signals of each BAC clone.

The CAPweb (Copy number Array analysis Platform on the web) platform developed by Institut Curie [14] was used for normalization (MANOR package), gain, loss or normal clone status assignment and breakpoint detection (GLAD package). The following default filtering parameters were retained: signal to noise ratio less than 3, standard deviation of replicates greater than 0.1 and exclusion of clones with missing values in over 50% of the tumors. Graphical representation of genomic alterations was performed with VAMP software (Institut Curie, Paris, France) [15,16]. The definition of gains and losses was based on median Cy3 to Cy5 log ratios greater than 1.2 and less than 0.8 respectively. Amplicons were defined by median log ratios greater than 2. The array CGH data are available in the ArrayExpress database (accession number: E-TABM-854) [9] or in the CIT database [10].

### Statistical analyses

Except where indicated, all transcriptomic analyses were carried out using R packages [17,18] or original R code. Raw gene expression data were normalized using the robust multi-array average (RMA) method from the R package *affy *[19]. Probe sets for control genes and those for which the 90th percentile of the log_2 _intensity was < 10 were removed, yielding a total of 48,927 probe sets.

#### Unsupervised classification of samples

**Figure 1:**
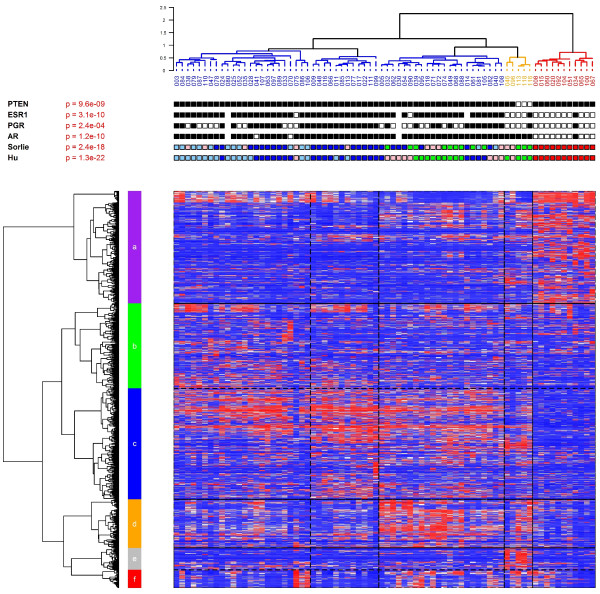
**Unsupervised hierarchical clustering of familial breast cancers**. Unsupervised hierarchical clustering using 2,447 highly variable probe sets. The orange branch contains the molecular apocrine tumors and includes the three Cowden tumors. PTEN: Germline *PTEN *mutation status (black box: wild type; white box: mutated). ESR1, PGR, AR: estrogen, progesterone and androgen receptor status determined by immunohistochemical staining [black box: positive (that is, score 2); white box: negative (that is, score 0 and 1 - see material and methods)]. Sorlie: The colored boxes represent the Stanford intrinsic gene classification based on the centroids described by Sorlie *et al*. [4] (*red*, basal-like class; *pink*, *HER2 *class; *green*, normal-like class; *dark blue*, luminal A class; *light blue*, luminal B class). Hu: same classification based on the revised centroids [2].

Probe sets were selected for clustering based on the following criteria: (i) *P*-value of a variance test less than 0.01 and (ii) a robust coefficient of variation (rCV) less than 10 but greater than the 95^th ^percentile of the rCV. For the variance test, we selected probe sets (P) whose variance across the samples was different from the median of the variances (Varmed) of all the probe sets. The statistic used was (n-1)´Var(P)/Varmed, where n refers to the number of samples. This statistic was compared to a percentile of the Chi-square distribution with (n-1) degrees of freedom and yielded a *P*-value for each probe set. This criterion is the same as that used in the filtering tool of the BRB ArrayTools software [20]. The rCV was calculated as follows: having ordered the intensity values of the n samples from minimum to maximum, we eliminated the minimum and maximum values and calculated the coefficient of variation for the remaining values. After filtering, we were left with 2,447 probe sets which were used for agglomerative hierarchical clustering using Ward's linkage and 1-Pearson correlation as a distance metric. The resulting dendrogram is used to order the genes and samples in the heatmap in Figure 1.

#### Assignment of samples to Stanford classes

In order to assign the tumor samples to the five subtypes in the Stanford classification, we calculated the Pearson correlation between each sample and each of the five centroids [4,21]. The probe sets in the two datasets were matched based on the UniGene IDs, resulting in a total of 334 common genes. Samples were then assigned to the subtype of the centroids with the largest correlation coefficient; this procedure is generally referred to as *Sorlie's centroid prediction*. The same procedure was applied to assign the tumor samples to the five subtypes in the Hu *et al*. classification [2], with a set of 213 common genes.

The Cowden signature was obtained by applying a Welch T-test comparing the samples in the Cowden cluster to all the other samples for the 48,927 probe sets retained after filtering. We selected the 3,075 probe sets with a *P*-value < 0.01 corresponding to a local False-Discovery-Rate of 20% (R package *kerfdr *[22]), then applied a random-forest procedure to choose the 200 probe sets best able to divide the samples into two groups, with a *P*-value < 0.001 (R package *randomForest *[23]) These 200 probe sets are the Cowden signature probe sets.

#### Gene set analysis

To define gene sets for pathway analysis, we mapped the biological pathway-related genes, gene ontology (GO) term-related proteins and public gene signatures to non-redundant Entrez Gene identifiers. For each GO term, we obtained a non-redundant list of protein identifiers, either directly associated with the GO term or one of its descendants, and mapped it to a non-redundant list of Entrez Gene ids, GO terms and their relationships (parent/child) [24]. Public signatures were downloaded from the molecular signature database MSigDB [25,26]. We used the hypergeometric test to measure the association between the Cowden signature (Entrez Gene ids) and a biological pathway, a GO term or a public signature, as described in the GOstats R package.

#### Principal components analysis

The first two principal components were used to visualize the tumors (*ade4 *R [27]). The groups of samples identified visually were confirmed by model-based clustering (R package *mclust *[28]). The signature showing transcriptomic differences between Cowden and non-Cowden molecular apocrine *carcinomas *was obtained by applying a Welch T-test to the three Cowden tumors and the two apocrine non-Cowden tumors following the same approach as that for the determination of the Cowden signature.

#### Statistical tests

Association of the sample subgroups to bio-clinical factors and to immunohistochemical factors was evaluated by applying a chi-square test (or the equivalent Fisher-exact test when appropriate).

The genomic rate of perturbation was defined as the mean, by chromosomal arm, of the ratio between the number of clones lost or gained and the total number of informative clones for each arm.

## Results

### Cowden tumors cluster together

The heatmap obtained after unsupervised clustering of the gene expression data from 74 tumors from patients with a familial clustering of breast cancer is shown in Figure 1. The major branches in the dendrogram correspond to the luminal A, luminal B and basal-like classes in the Stanford classification [4] (*P *= 2.4 × 10^-18 ^with the Sorlie intrinsic gene set and *P *= 1.3 × 10^-22 ^with the Hu intrinsic gene set). Interestingly, the three Cowden tumors, along with two supplementary tumors, lie on a separate branch, marked orange in the figure, characterized by over-expression of a group of about 84 genes (cluster e, Figure 1). Correlation with the Stanford centroids [4] was used to classify these tumors. Two were assigned to the HER2 class and three to the normal-like class, indicating that the observed clustering can not be explained by the Stanford model. The two non-Cowden tumors were tested for somatic point mutations and large rearrangements in the PTEN gene, but no abnormalities were detected.

### The Cowden signature is similar to a molecular apocrine signature

**Figure 2:**
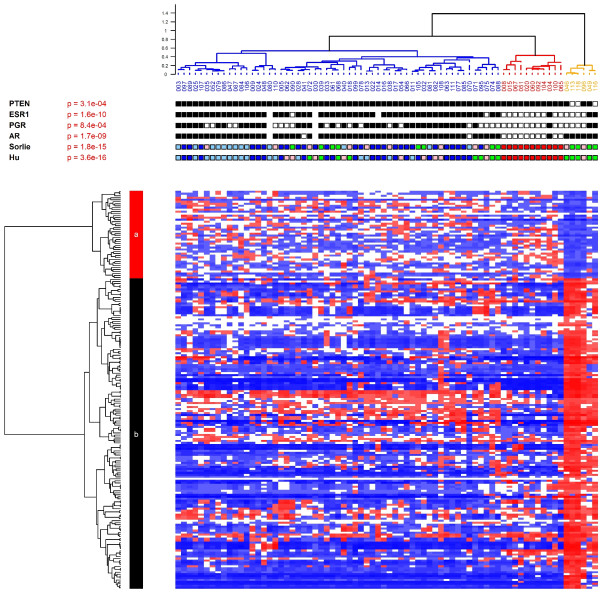
**Supervised hierarchical clustering of familial breast cancers**. Hierarchical clustering using 200 probe sets that distinguish the orange branch from the other tumors in Figure 1. Table S1 in Additional file 1 lists the corresponding genes. PTEN, ESR1, PGR, AR, Sorlie, Hu: see Figure 1.

**Figure 3:**
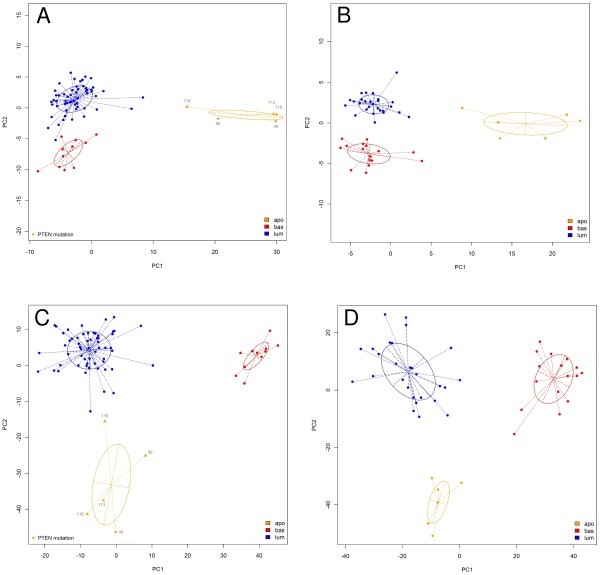
**Principal components analysis using the Cowden and molecular apocrine (Farmer) signatures (13)**. The first two principal components were used to plot the tumors from this study in **A **and **C**, and to plot the tumors from the Farmer study in **B **and **D**. The genes used were derived from the Cowden signature in A and B, and from the Farmer signature in C and D. In both cases, the signatures from the two studies identify the same tumors.

To understand the molecular basis for this Cowden-specific clustering, a supervised analysis was performed (Figure 2) identifying a molecular signature of 101 overexpressed genes and 30 underexpressed genes in the Cowden group (Table S2 in Additional file 1). Pathway and Gene Ontology analyses of the Cowden tumors (Table 1) showed enrichment for genes belonging to several metabolic pathways, including lipid metabolism (*P *= 1 × 10^-10^), PPARγ signaling (*P *= 3 × 10^-6^) and androgen and estrogen metabolism (*P *= 1 × 10^-4^). Comparison with published data [29] showed that there was no overlap between our signature and the Agendia *70-gene profile *[30] (70 probes) or the OncotypeDx *recurrence score *[31] (16 genes), and there was only minimal overlap with the *Core Serum Response *gene set [32] (5 genes in common out of 416 probes). The enrichment in metabolic genes in the Cowden signature is reminiscent of *molecular apocrine tumors *[33], a tumor type suspected to have increased androgenic signaling that overlaps with the HER2 class in the Stanford classification. In total, 54 genes are shared by the Cowden (131 genes) and apocrine (556 genes) signatures [33]. This overlap is highly significant (*P *= 6 × 10^-50^) and substantially greater than with any other signatures we tested (Table 1). A principal components analysis (PCA) (Figure 3) with the genes of the Farmer signature [33] showed that the tumors of our study fall into three discrete groups containing the basal-like, the luminal and the Cowden-like tumors (Figure 3C). The converse is also true: principal components analysis of the Farmer tumors with the Cowden genes identified six Cowden-like tumors corresponding to the six tumors previously labeled as molecular apocrine subtype (Figure 3B). We conclude that the Cowden and molecular apocrine signatures identify the same tumors.

**Table 1:** Gene ontology enrichment for the Cowden signature and comparison with other signatures

Gene Ontology interrogation	*P*-value
Oxidoreductase activity	3 × 10^-11^
Lipid metabolic process	1 × 10^-10^
Cellular lipid metabolic process	4 × 10^-7^
Carboxylic acid metabolic process	1 × 10^-5^
Organic acid metabolic process	1 × 10^-5^
	
**KEGG pathway database interrogation**	
PPAR signaling pathway	3 × 10^-6^
Bile acid biosynthesis	6 × 10^-5^
Androgen and estrogen metabolism	1 × 10^-4^
Arachidonic acid metabolism	2 × 10^-4^
Metabolism of xenobiotics by cytochrome P450	4 × 10^-4^
	
**MSig database interrogation**	
Molecular apocrine signature (Farmer *et al*.)	6 × 10^-50^
Genes upregulated by androgen in neoplastic prostate epithelium (Nelson *et al*.)	1 × 10^-10^
Genes annotated in NetAffx as androgen related (NetAffx)	2 × 10^-8^
Genes downregulated in AIDS-related primary effusion lymphoma (PEL) cells compared to normal B cells and other tumor subtypes. (Klein *et al*.)	2 × 10^-4^
Genes downregulated by telomerase (Smith *et al*.)	4 × 10^-4^
Downregulated in mature, differentiated adipocytes following treatment with TNFalpha (Ruan *et al*.)	4 × 10^-4^

### GGT1 is an immunohistochemical marker for sporadic and Cowden disease breast cancers with apocrine profile

**Figure 4:**
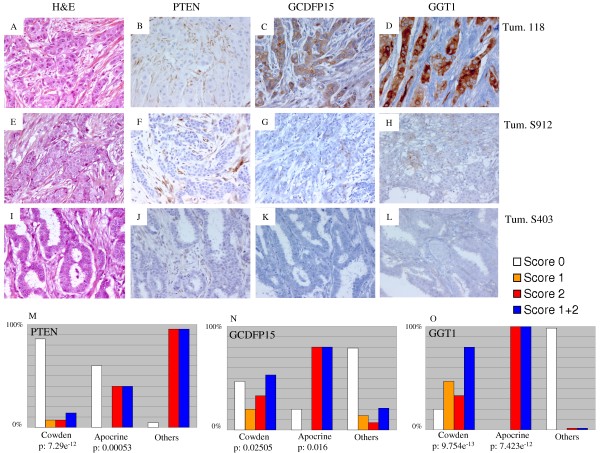
**Immunohistochemical analysis of the whole panel of breast tumors**. Sections of three breast tumors occurring in patients with Cowden disease showing negative staining for PTEN **(B, F, J)**, positive staining scored 2 for GCDFP15 **(C) **and GGT1 **(D)**, positive staining scored 1 for GCDFP15 **(G) **and GGT1 **(H) **and negative staining for GCDFP15 **(K) **and GGT1 **(L)**. The proportion of tumors showing positive staining for these proteins in Cowden tumors (*n *= 15), molecular apocrine tumors (*n *= 5) and the remaining familial breast cancers (*n *= 69) is shown in (**M **to **O**). See material and methods for attribution of scores 0, 1 and 2.

To test whether apocrine differentiation is a general feature of Cowden disease breast cancers, we examined an additional 12 tumors, for which RNA was not available, by histological and immunohistochemical techniques on a tissue microarray containing the 12 new and the 74 original tumors. To identify antibodies that work on formalin-fixed paraffin-embedded material we screened for antibodies showing the largest difference in mean expression level between the Cowden/apocrine tumor group and the others. The most discriminating antibody was against gamma-glutamlytransferase (GGT1). We also assessed expression of the androgen receptor (AR) and the epidermal growth factor receptor (EGFR) because of their association with the molecular apocrine subtype [33,34], GCDFP15 (*PIP*), a marker of apocrine differentiation currently used in routine pathological practice [33,35], PTEN itself, and the classic markers ERBB2, estrogen receptor alpha (ER) and progesterone receptor (PR). The results are summarized in Table 2 and detailed in Tables S3 and S4 (Additional file 1). In the initial set of tumors, three have histological features of classic apocrine carcinoma (cases 46, 113 and 118), one is an invasive ductal carcinoma with apocrine features (case 96), and the remaining tumor is a poorly differentiated invasive ductal carcinoma without apocrine features (case 116). All of the tumors in this group are strongly positive for GGT1 (Figure 4), and the four tumors with histological apocrine features expressed EGFR (cases 46, 96, 113 and 118). In the new set of Cowden tumors, two have histological features of classic apocrine carcinoma (cases 295 and 891) and are GGT1 positive. Two are invasive ductal (cases 681 and 732), one is a micro papillary carcinoma (case 712), all with apocrine features that do not meet the full criteria for apocrine carcinoma; all three are weakly positive for GGT1. Overall, if one uses a threshold of 1% of GGT1-stained cells to score tumors as positive, 12 out of the 15 Cowden tumors are positive for GGT1 and only one of 69 in the control group (*P *= 9 × 10^-13^) (Figure 4). GCDFP15 was less useful as a Cowden or apocrine marker since 8 of the 15 Cowden tumors were positive, compared with 14 of the 69 control tumors (*P *= 0.025) (Table 2, Tables S3 and S4 in Additional file 1, and Figure 4). The other markers showed that typical invasive apocrine carcinomas are AR-positive, ER-negative and EGFR positive. The Cowden tumors were all AR-positive but only 27% were ER-negative, indicating that the phenotype of apocrine carcinomas is not identical to that of Cowden breast cancers. Interestingly, PTEN immunohistochemistry was negative in 13 of the 15 Cowden tumors, compared to only 3 of 69 in the control group. The two non-Cowden molecular apocrine tumors showed clear PTEN positivity. We conclude that breast cancers occurring in women with Cowden disease commonly show apocrine differentiation and that GGT1 appear to be a useful marker to identify molecular apocrine carcinomas.

**Table 2:** Characteristics of the Cowden breast cancers and the non-Cowden apocrine carcinomas

Tumor sample	Cowden disease	*PTEN *germline mutation status	Age at diagnosis of cancer	Histologic type	Histologic grade	Apocrine features	AR	ER	PR	ERBB2	GCDFP15	GGT1	PTEN
46	No	WT	60 y	IAC	2	Yes	2	0	0	0	2	2	2
96	No	WT	40 y	IDC	3	Yes	2	0	0	+++	2	2	2
113	Yes	c.209+5G > A	48 y	IAC	1	Yes	2	0	0	0	2	2	0
116	Yes	c.1007dupA p.Tyr336X	44 y	IDC	2	No	2	2	2	0	0	2	0
118	Yes	c.209+5G > A	35 y	IAC	2	Yes	2	0	0	0	2	2	0
S89	Yes	c.158_159insATAC p.val54TyrfsX10	44 y	IDC	2	No	2	2	2	0	0	0	0
S243	Yes	c.323T > C p.Leu108Pro	28 y	IDC	2	No	2	2	2	0	0	1	0
S295	yes	c.209+5G > A	44 y	IAC	2	Yes	2	0	0	0	2	2	0
S362	Yes	c.69dupA p.Asp24ArgfsX20	53 y	DCIS	low	No	2	2	2	0	0	1	0
S403	Yes	c.801+1delG	43 y	IDC	2	No	2	2	2	0	0	0	0
S574	Yes	c.491delA p.Lys164ArgfsX3	46 y	IDC	1	No	2	2	2	0	0	1	0
S681	Yes	c.830C > G p.Thr277Arg	27 y	IDC	3	Yes	2	2	1	+	1	1	0
S712	Yes	c.592delA p.Met198X	41 y	MPC	2	Yes	2	2	0	nd	0	1	0
S730	Yes	c.493G > A p.Gly165Arg	59 y	ILC	2	No	2	2	2	+	2	0	0
S732	Yes	c.510T > G p.Ser170Arg	46 y	IDC	2	Yes	2	2	2	0	1	1	1
S891	yes	c.209+5G > A	35 y	IAC	2	Yes	2	0	0	++	2	2	2
S912	Yes	c.632_633delGC p.Cys211X	34 y	IDC	2	No	2	2	2	0	1	1	0

IHC status excepted for ERBB2 (0 to +++): 0 = negative; 1 = positive up to 10% of cells; 2 = positive above 10% of cells. DCIS, ductal carcinoma in situ; IAC, invasive apocrine carcinoma; IDC, invasive ductal carcinoma; ILC, invasive lobular carcinoma; MPC, micro papillary carcinoma; nd, not determined; WT, wild type.

### Molecular apocrine carcinomas have genetic alterations of the ERBB2/PIK3CA/PTEN pathway

**Figure 5:**
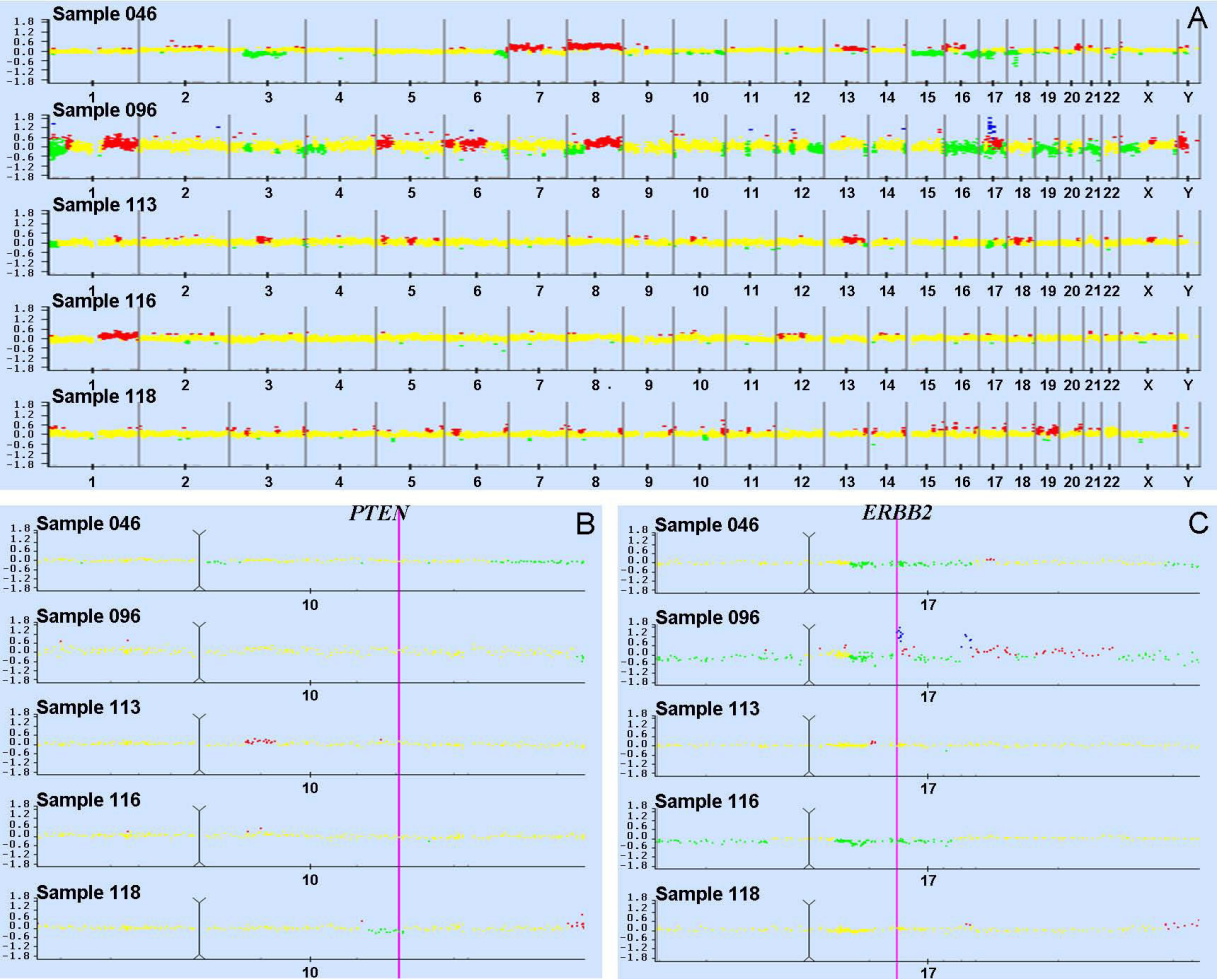
**Array CGH of the five molecular apocrine tumors**. Graphical view (VAMP software) showing the log2 ratio of the fluorescence intensities for each clone. Gains appear in red, amplicons in blue, losses in green and balanced signals in yellow. **(A) **Pan genomic array CGH profile of the Cowden (*n *= 3) and non-Cowden (*n *= 2) molecular apocrine tumors. **(B) **Plot of chromosome 10 showing genomic loss in sample 118 at the *PTEN *locus. The pink bar and the black line indicate respectively the *PTEN *locus and the centromere position. **(C) **Plot of chromosome 17 showing amplicons in sample 96. The proximal amplicon at 17q12 contains the *ERBB2 *gene as indicated by the pink bar.

The main genetic lesion associated with molecular apocrine carcinoma in the literature is *ERBB2 *amplification [33,36,37]. To identify additional lesions, we performed array CGH of the five tumors showing a molecular apocrine profile including the three Cowden tumors. The results are shown in Figure 5. In contrast with typical sporadic breast cancers, the CGH profiles of molecular apocrine Cowden tumors show no major abnormalities, with a genomic perturbation rate of 0.08, 0.05 and 0.12, respectively, for tumors 113, 116 and 118, while apocrine non-Cowden tumors show a higher rate of 0.27 and 0.43, respectively, for tumors 46 and 96 (Table S5 in Additional file 1). By comparison, the mean value of perturbation rate in a series of 135 unselected breast carcinomas is 0.28 (range 0.02 to 0.73) (unpublished data). Specifically, gain or loss of large segments of chromosome arms, a defect very commonly seen in sporadic tumors, was only seen in three tumors, and was restricted to chromosomes, 1, 8, 15, 16, 19 and 21 (Figure 5A). On the other hand, many small gains and losses were observed particularly affecting chromosome 17. Only one tumor (case 96) had an amplicon on chromosome 17 that included the *ERBB2 *gene (Figure 5C). Interestingly, this was one of the two non-Cowden tumors in the molecular apocrine group. One obvious question is whether the CGH profiles for the Cowden tumors show loss of heterozygosity for the *PTEN *locus. The small genomic rearrangements observed in tumor 118 include a specific loss at the chromosomal band 10q23 that spans the *PTEN *gene (Figure 5B). Sequencing of the corresponding tumor DNA confirmed that the deletion eliminates the wild type *PTEN *allele in that tumor. Although a slight predominance of the mutated allele is observed for tumor 116, no clear evidence for allelic loss was obtained from sequencing and semi quantitative multipex PCR for the two other Cowden tumors (data not shown). It is however difficult to definitively conclude the PTEN allelic status because of normal cell contamination that can mask a loss of genetic material in tumoral cells. Evidence for the loss of the wild type allele in at least one tumor suggests that *PTEN *behaves like a classic tumor suppressor gene in Cowden tumors. Further support for this model comes from immunohistochemical staining for PTEN, which was reduced or absent in all but the two non-Cowden tumors in the panel tested (Table 2; Figure 4). The loss of the PTEN protein expression associated with the probable retention of the wild type *PTEN *allele in cases 113 and 116 suggests that additional changes, such as DNA methylation, may eliminate expression of the wild type allele in some cases.

### Transcriptomic differences between Cowden tumors and molecular apocrine carcinomas

Given the relatively flat CGH profiles of the Cowden tumors, which contrast strongly with the expected, amplicon-rich profiles in sporadic molecular apocrine tumors, we performed a supervised analysis of gene expression data to identify potential explanations for the difference between the two tumor types. The results, which should be treated with caution because of the small number of tumors tested, are given in Table S6 and S7 (Additional file 1) and in Figure S1 in Additional file 2. We identified a signature of 200 probe sets corresponding to 54 genes upregulated in Cowden breast tumors and 110 genes upregulated in non-Cowden apocrine tumors. Pathway and Gene Ontology enrichment analyses indicated that the Cowden tumors preferentially express genes involved in MAPK and JAK-STAT signaling pathways whereas non-Cowden apocrine tumors express genes involved in insulin and calcium signaling pathways.

## Discussion

The main conclusion from this study is that germline *PTEN *mutation predisposes to the formation of breast tumors with apocrine features. This is intriguing because of the large body of work linking ERBB2, PTEN and PI3-kinase signaling to breast cancer. Interestingly, the timing of PTEN loss during tumorigenesis seems to dictate the phenotype, with apocrine features particularly associated with germline PTEN loss.

In the original microarray-based classification of breast cancer, Perou and colleagues identified a group of tumors enriched in ERBB2-amplified tumors that they called the HER2 class [1]. Further analysis of this group on Affymetrix gene expression arrays, which have a broader selection of genes than was available to Perou, confirmed the observation that *ERBB2 *is commonly amplified [1,3,4], but we proposed the name *molecular apocrine *for the group to reflect the RNA phenotype [33] and the fact that many ERBB2-amplified tumors lie outside this group. The results of the present study confirm the existence of a molecular apocrine group, and further weaken the argument for using HER2 to label it, because the tumors with this phenotype showing germline PTEN mutations lack ERBB2 amplification.

Apocrine carcinoma is a classic histological subtype of breast carcinoma with characteristic morphological and immunohistochemical features [38,39]. It belongs to a spectrum of apocrine metaplasia including common benign conditions like fibrocystic disease that are associated with increased androgen signaling. In general, the link with androgens is stronger at the benign end of the spectrum, but some malignant tumors in the apocrine group also show increased expression of androgen receptor target genes, and a breast cancer cell line (MDA-MB-453) with a molecular apocrine gene expression profile was recently shown to be androgen-dependent for growth [36]. Interestingly, this cell line has an inactivating PTEN mutation (c.919G > A - p.Glu307Lys) [40]. Classic apocrine carcinoma is not known to be linked to Cowden disease, but benign mammary lesions occurring in Cowden disease frequently show apocrine differentiation [41,42], and 4 of the 15 tumors in our series met the full histological criteria for a diagnosis of apocrine carcinoma. The original molecular apocrine description linked this subtype of tumors to the AR positive and ER negative phenotype. In this series, several Cowden tumors, including tumor 116 that belongs to the Cowden/apocrine transcriptomic cluster are ER positive. This tumor may be an example of the recently described apocrine-like carcinoma which includes AR and ER positive tumors [34]. Additional transcriptomic studies of Cowden breast carcinoma will be necessary to confirm and extend this hypothesis.

The two non-Cowden apocrine carcinomas in this study express a normal level of wild type *PTEN*, indicating that some other mechanism must explain the apocrine differentiation of these tumors. Interestingly, one of these tumors has amplified the *ERBB2 *gene and the other has a *PIK3CA *mutation (c.3140A > G; p.His1047Arg - data not shown). Thus, all five apocrine tumors for which we have RNA, DNA and immunohistochemical data show a specific genetic alteration in the ERBB2-PTEN-PIK3CA pathway. This observation seems to indicate that if the main phenotypic trait of apocrine carcinomas is increased androgen signaling, the main genetic trait is mutation of genes in the ERBB2-PTEN-PIK3CA pathway. The number of tumors is too small to draw a definitive conclusion but the observation is provocative and deserves further study.

The link between *PTEN *and apocrine differentiation seems not to extend to somatic *PTEN *mutations. Although rare, somatic *PTEN *mutations have previously been reported in breast cancer [43,44], but they were seen mainly in the basal-like tumors class characterized by a loss of expression of PTEN [45] and more specifically in tumors from patients with *BRCA1 *mutations [40]. The 74 familial breast cancers microarrayed in this study include seven linked to *BRCA1 *and five linked to *BRCA2*. None of these tumors has a molecular apocrine profile. In the same way, tumors 9, 11 and 15 have lost PTEN expression (Table S2 in Additional file 1) but do not have a molecular apocrine profile (luminal A for tumors 9 and 11; basal-like for tumor 15). Thus, somatic *PTEN *mutation appears not to be directly linked to apocrine differentiation in our panel of familial tumors. To formally exclude a role in sporadic tumors, a specific study of *PTEN *alterations in sporadic apocrine carcinoma should be done.

The mechanism leading from germline *PTEN *mutation to apocrine differentiation is unknown, but there are numerous hints in the literature. First, PTEN has been shown to inhibit androgen receptor-driven transcription in LNCaP prostate cancer cells in an AKT-dependent or independent manner [46,47]. Loss of *PTEN *in this model increases androgen signaling, which is itself a feature of apocrine cells. Second, *ERBB2 *amplification is commonly seen in sporadic tumors in the molecular apocrine class. ERBB2 has been shown to stabilize AR protein in prostate cancer cells [48] and to activate the Akt pathway [49]. Third, in a large-scale RNA interference screen, PTEN silencing conferred resistance to trastuzumab in breast cancer cells with *ERBB2 *amplification [50]. Fourth, PTEN-deficient/Erbb2^KI ^transgenic mice show accelerated mammary tumor onset associated with elevated ERBB2 protein levels that are not caused by *ERBB2 *amplification [51]. A subset of the mouse tumors has large nuclei, prominent nucleoli and abundant cytoplasm, the hallmarks of apocrine histology. This confirms that increased ERBB2 signaling in the context of germline *PTEN *loss predisposes to apocrine tumorigenesis, and provides a transgenic mouse model to study apocrine breast cancer. Finally, the Gene Ontology analysis in Table 1 links the Cowden signature to increased PPARγ signaling. PPARγ has previously been linked with the PTEN pathway [52], it transactivates the PTEN promoter [53], and PPARγ agonists increase PTEN expression in breast cancer cell lines. Activation of the PPARγ pathway following loss of PTEN could be explained by negative feedback of PTEN on PPARγ.

## Conclusions

Taken together, our results in Cowden disease and multiple results in the literature strongly support a central role for the ERBB2-PTEN-PIK3CA-AKT pathway in the biology of breast cancer. We suspect that loss of PTEN early in tumorigenesis leads to the expansion of a cell in the hierarchy of mammary differentiation with a pronounced tendency to undergo apocrine metaplasia. Current models for mammary differentiation would place this cell at a late point in the hierarchy, after the luminal progenitor stage [54].

## Abbreviations

AR: androgen receptor; CY5: cyanine 5; CY3: cyanine 3; EGFR: epidermal growth factor receptor; ER: estrogen receptor; GO: gene ontology; P: probe sets; PCA: principal components analysis; RCV: robust coefficient of variation; RMA: robust multi-array average; Varmed: median of the variances.

## Competing interests

The authors declare that they have no competing interests.

## Authors' contributions

ML, FB and NS planned and supervised the work. GB performed the experiments, except for immunohistochemistry. GB and RS managed the data. VV performed TMA and immunohistochemistry experiments. IDM and GMG read immunohistochemistry slides. FC, VB, AD, CD, BG, OI and PV provided samples from Cowden patients with breast cancer. MG, GB, ADR and RI performed statistical and bioinformatic analyses. GB, GMG, MG, RI and ML wrote the manuscript.

## Acknowledgements

We thank the patients and their families who contributed to this study. We thank Laurent Arnoult, Catherine Clement-Chassagne, Sophie Guymar, Pierre Le Villain, Beaudoin Mazet, Brigitte Sigal-Zafrani, Patrick Tass and Jean-Laurent Totobenazara for providing paraffin-embedded tumor blocks. The expertise of the members of the Affymetrix expression array platform at IGBMC, Strasbourg is gratefully acknowledged.

This work is part of the national program "Cartes d'Identité des Tumeurs" [10] funded and developed by the "Ligue Nationale contre le Cancer". We thank the Charente Maritime Cancer League, the Pyrenees Atlantiques Cancer League, the Canceropole Grand Sud-Ouest (ACI 2004 renewed 2007) and the Bergerac Lions Club for funding to ML. We thank the French Foundation for Medical Research (FRM) and the Bergonié Cancer Institute for funding to GB.

## Supplementary Material

Additional file 1 **Supplementary tables**. Table S1: Major clinical characteristics of the studied tumors. Table S2: List of the probe sets constituting of the Cowden signature. Table S3: Detailed results for immunohistochemistry study from the 74 initial tumors and the 12 supernumerary Cowden breast cancers indicating the percentage of immunoreactive tumor cells and the intensity of a positive signal. Stanford classification (with Sorlie and Hu intrinsic gene set) for each tumor with RNA available is also indicated. Table S4: Immuno-histo-chemistry scoring for the three categories of tumors: Cowden tumors (*n *= 15), molecular apocrine tumors (*n *= 5) and the remaining familial breast cancers (*n *= 69). Table S5: array-CGH derived rate of perturbation and percentage of tumor cells evaluated for the five molecular apocrine tumors. Table S6: List of probe sets distinguishing between Cowden and non-Cowden tumors within apocrine breast cancers. Table S7: Gene ontology and KEGG pathway database interrogation by over expressed genes in Cowden and non Cowden apocrine breast cancers. Click here for file

Additional file 2 **Supplementary figures**. Figure S1: Hierarchical clustering using 200 probe sets that distinguish Cowden from non Cowden apocrine breast cancers. Click here for file
