# Supplementary material for: Molecular apocrine differentiation is a common feature of breast cancer in patients with germline PTEN mutations
Source: Breast Cancer Res. 2010 Aug 16;12(4):R63. doi: 10.1186/bcr2626 (PMC2949656; doi:10.1186/bcr2626)
Supplement: Additional file 1 — Supplementary tables. Table S1: Major clinical characteristics of the studied tumors. Table S2: List of the probe sets constituting of the Cowden signature. Table S3: Detailed results for immunohistochemistry study from the 74 initial tumors and the 12 supernumerary Cowden breast cancers indicating the percentage of immunoreactive tumor cells and the intensity of a positive signal. Stanford classification (with Sorlie and Hu intrinsic gene set) for each tumor with RNA available is also indicated. Table S4: Immuno-histo-chemistry scoring for the three categories of tumors: Cowden tumors (n = 15), molecular apocrine tumors (n = 5) and the remaining familial breast cancers (n = 69). Table S5: array-CGH derived rate of perturbation and percentage of tumor cells evaluated for the five molecular apocrine tumors. Table S6: List of probe sets distinguishing between Cowden and non-Cowden tumors within apocrine breast cancers. Table S7: Gene ontology and KEGG pathway database interrogation by over expressed genes in Cowden and non Cowden apocrine breast cancers. [file bcr2626-S1.PDF]

Table S1: Major clinical, pathological and genealogical characteristics of the studied patients

Tumor Sample ID	Age at diagnosis of cancer (year)	Sex	Histologic type	Histologic grade	Relatives degree	Number of familial breast cancers	Average age of familial breast cancers	Number of others familial cancers
_003	37	F	Invasive ductal carcinoma	3	1	2	45	0
_005	69	F	Invasive ductal carcinoma	1	1	2	61	2
_008	51	F	Invasive ductal carcinoma	3	1	2	37	3
_009	51	F	Invasive ductal carcinoma	3	1	3	49	1
_011	56	F	Invasive ductal carcinoma	2	1	4	52	0
_013	41	F	Invasive ductal carcinoma	2	2	3	62	1
_014	45	F	Invasive ductal carcinoma	2	1	3	45	0
_015	42	F	Invasive ductal carcinoma	3	2	3	51	3
_016	49	F	Invasive ductal carcinoma	3	2	4	53	0
_017	76	F	Invasive ductal carcinoma	1	1	3	70	0
_018	60	F	Invasive ductal carcinoma	2	1	3	59	2
_020	59	F	Invasive ductal carcinoma	3	1	6	41	0
_022	64	F	Invasive ductal carcinoma	2	1	4	66	2
_024	35	F	Invasive ductal carcinoma	1	2	2	37	3
_025	37	F	Invasive ductal carcinoma	2	1	3	50	0
_028	68	F	Invasive ductal carcinoma	1	1	2	60	1
_030	51	F	Invasive ductal carcinoma	nd	1	3	42	2
_032	44	F	Invasive ductal carcinoma	2	2	8	42	0
_033	43	F	Invasive ductal carcinoma	1	1	2	50	0
_034	36	F	Invasive ductal carcinoma	3	1	3	50	3
_035	49	F	Invasive ductal carcinoma	2	2	2	38	0
_038	64	F	Invasive ductal carcinoma	3	1	5	58	1
_039	32	F	Invasive ductal carcinoma	2	1	2	40	2
_040	38	F	Invasive ductal carcinoma	2	1	2	38	1
_041	41	F	Invasive ductal carcinoma	3	1	2	45	0
_046	61	F	Invasive apocrine carcinoma	2	1	3	64	0
_047	54	F	Invasive ductal carcinoma	3	2	2	53	2
_048	54	F	Invasive ductal carcinoma	1	1	2	45	0
_049	50	F	Invasive ductal carcinoma	2	1	2	45	0
_051	43	F	Adenocarcinoma with spindle cell metaplasia	3	2	3	39	1
_052	35	F	Invasive ductal carcinoma	3	1	2	40	5
_054	73	F	Invasive lobular carcinoma	2	1	4	60	2
_060	41	F	Invasive ductal carcinoma	3	1	2	46	1
_061	35	F	Invasive ductal carcinoma	1	2	3	43	0
_062	51	F	Invasive lobular carcinoma	2	2	2	46	0
_063	38	F	Invasive ductal carcinoma	1	1	2	46	1
_065	46	F	Invasive ductal carcinoma	3	1	2	32	0
_066	73	F	Invasive ductal carcinoma	2	1	4	59	2
_067	36	F	Invasive ductal carcinoma	3	1	2	36	0
_068	48	F	Invasive ductal carcinoma	1	2	3	58	0
_070	39	F	Invasive ductal carcinoma	3	1	5	53	3
_071	44	F	Invasive ductal carcinoma	2	2	8	42	0
_072	39	F	Invasive ductal carcinoma	2	1	2	43	0
_074	48	F	Invasive ductal carcinoma	2	2	3	46	0
_075	58	F	Invasive ductal carcinoma	2	1	2	52	2
_077	53	F	Invasive ductal carcinoma	1	2	3	56	1
_078	55	F	Invasive ductal carcinoma	2	1	2	40	2
_079	41	F	Invasive ductal carcinoma	3	1	2	37	0
_080	33	F	Invasive ductal carcinoma	nd	1	4	42	0
_081	46	F	Infiltrating duct and Invasive lobular carcinoma	2	2	6	52	0
_082	49	F	Invasive ductal carcinoma	1	1	2	50	3
_084	47	F	Invasive ductal carcinoma	3	1	3	53	4
_085	64	F	Invasive ductal carcinoma	1	1	3	63	1
_086	47	F	Invasive ductal carcinoma	3	1	1	46	4
_087	68	F	Invasive ductal carcinoma	3	1	1	67	5
_088	52	F	Invasive ductal carcinoma	1	1	2	36	1
_089	30	F	Invasive ductal carcinoma	nd	2	4	39	1
_090	40	F	Invasive lobular carcinoma	3	1	1	39	3
_092	52	F	Invasive ductal carcinoma	3	2	4	56	0
_095	77	F	Invasive lobular carcinoma	1	1	2	61	6
_096	41	F	Invasive ductal carcinoma	3	1	3	51	1
_097	45	F	Invasive ductal carcinoma	3	1	3	57	1
_099	63	M	Invasive ductal carcinoma	1	1	3	54	0
_100	57	F	Invasive ductal carcinoma	3	1	5	54	2
_104	44	F	Invasive ductal carcinoma	3	1	3	38	2
_105	57	F	Invasive ductal carcinoma	2	1	5	56	0

Table S1: Major clinical, pathological and genealogical characteristics of the studied patients

Tumor Sample ID	Age at diagnosis of cancer (year)	Sex	Histologic type	Histologic grade	Relatives degree	Number of familial breast cancers	Average age of familial breast cancers	Number of others familial cancers
_106	57	F	Invasive ductal carcinoma	3	1	2	37	0
_107	70	F	Mucinous adenocarcinoma	1	1	2	68	4
_108	44	F	Invasive ductal carcinoma	3	1	2	45	3
_110	29	F	Invasive ductal carcinoma	2	1	5	46	0
_111	73	F	Invasive ductal carcinoma	1	1	3	68	0
_113	49	F	Invasive apocrine carcinoma	1	1	3	48	4
_116	44	F	Invasive ductal carcinoma	2	1	1	44	0
_118	35	F	Invasive apocrine carcinoma	2	1	3	48	4
_S89	44	F	Invasive ductal carcinoma	2	nd	nd	nd	nd
_S243	28	F	Invasive ductal carcinoma	2	nd	nd	nd	nd
_S295	44	F	Invasive apocrine carcinoma	2	nd	nd	nd	nd
_S362	53	F	Ductal carcinoma in situ	low	nd	nd	nd	nd
_S403	43	F	Invasive ductal carcinoma	2	nd	nd	nd	nd
_S574	46	F	Invasive ductal carcinoma	1	nd	nd	nd	nd
_S681	27	F	Invasive ductal carcinoma	3	nd	nd	nd	nd
_S730	59	F	Invasive lobular carcinoma	2	nd	nd	nd	nd
_S712	41	F	Micro papillary carcinoma	2	nd	nd	nd	nd
_S732	46	F	Invasive ductal carcinoma	2	nd	nd	nd	nd
_S891	35	F	Invasive apocrine carcinoma	2	nd	nd	nd	nd
_S912	34	F	Invasive ductal carcinoma	2	nd	nd	nd	nd

Table S1: List of the probe sets constituting of the Cowden signature

pbs	gene symbol	chrom	arm	cytogenet	start	end	genes cluster	p-value	fold-change
1562447_a_at	---	9	9q	q34.3	137499803	137512385	a	2,87E-15	-0,43593369
226313_at	C10orf35	10	10q	q21.3	71060305	71063358	a	2,11E-11	-0,68192255
226567_at	USP14	18	18p	p11.32	203613	204736	a	4,06E-08	-0,6215581
1559780_at	DDAH1	1	1p	p22.3	85640452	85642528	a	4,57E-08	-0,58594099
219258_at	TIPIN	15	15q	q22.31	64416229	64432349	a	1,13E-07	-0,95913656
208002_s_at	ACOT7	1	1p	p36.31	6246918	6376025	a	1,23E-06	-1,07257101
213785_at	IPO9	1	1q	q32.1	200118536	200120042	a	1,57E-06	-0,83709371
202641_at	ARL3	10	10q	q24.32	104426304	104455237	a	3,36E-05	-0,97432274
1556144_at	DHX30	3	3p	p21.31	47819598	47859058	a	3,76E-05	-0,42369862
218911_at	YEATS4	12	12q	q15	68039798	68070842	a	0,00010517	-1,02630275
242056_at	TRIM45	1	1p	p13.1	117455206	117455860	a	0,00010894	-1,36787902
1568873_at	ZNF519	18	18p	p11.21	14089945	14122427	a	0,00015371	-0,99308184
228812_at	---	2	2p	p16.1	61011008	61012250	a	0,00015502	-1,13056842
207358_x_at	MACF1	1	1p	p34.3	39469437	39724699	a	0,00017633	-0,76163303
203983_at	TSNAX	1	1q	q42.2	229731021	229768890	a	0,00023845	-0,91002259
1569701_at	PER3	1	1p	p36.23	7769968	7771216	a	0,00025592	-1,24876164
235039_x_at	LIN9	1	1q	q42.12	224485699	224493359	a	0,0003203	-0,63218435
202916_s_at	FAM20B	1	1q	q25.2	177261653	177312320	a	0,00033176	-0,77253508
205283_at	FKTN	9	9q	q31.2	107377044	107443219	a	0,0003411	-0,87465438
226341_at	---	8	8q	q21.3	92149321	92150640	a	0,00034174	-1,00278391
1558369_at	MPHOSPH9	12	12q	q24.31	122204731	122206833	a	0,00035714	-0,99047975
204662_at	CP110	16	16p	p12.3	19442778	19472227	a	0,00041718	-0,9231633
235130_at	PANK2	20	20p	p13	3846209	3847286	a	0,00042177	-0,41204552
221909_at	RNFT2	12	12q	q24.22	115773636	115775819	a	0,00042971	-1,37986061
215222_x_at	MACF1	1	1p	p34.2	39689381	39724329	a	0,00047637	-0,63905773
225456_at	MED1	17	17q	q12	34814063	34816026	a	0,00048103	-0,77567642
212238_at	ASXL1	20	20q	q11.21	30481397	30490782	a	0,00048365	-0,66916179
237881_at	---	15	15q	q26.3	97140520	97140922	a	0,00049839	-2,05564923
242156_at	---	2	2p	p13.2	73673364	73673825	a	0,00050608	-0,99481906
214999_s_at	RAB11FIP3	16	16p	p13.3	451404	487449	a	0,0005258	-0,5926189
204299_at	FUSIP1	1	1p	p36.11	24168159	24179404	a	0,00056255	-0,61206443
232168_x_at	MACF1	1	1p	p34.2	39694964	39703169	a	0,00061882	-0,60719995
1557164_a_at	---	9	9q	q22.32	97684168	97684774	a	0,0006495	-0,67560609
226883_at	---	17	17q	q21.2	37258295	37259038	a	0,00073763	-0,73600563
242563_at	FCHSD2	11	11q	q13.4	72448422	72449258	a	0,00075447	-0,90799488
1559988_at	ZNF483	9	9q	q31.3	113327333	113346533	a	0,00079595	-0,232543
1564773_x_at	---	12	12q	q13.2	54468573	54987291	a	0,00080576	-0,61991063
206273_at	SLMO1	18	18p	p11.21	12421580	12422232	a	0,00081023	-0,71569851
242439_s_at	ASXL1	20	20q	q11.21	30410948	30411530	a	0,00081929	-0,94579813
215170_s_at	CEP152	15	15q	q21.1	46820636	46890519	a	0,0008326	-0,60719148
236780_at	---	9	9q	q33.3	126670329	126670793	a	0,0008996	-0,71026123
215905_s_at	WDR57	1	1p	p35.2	31505143	31515100	a	0,000944	-0,55092427
220078_at	USP48	1	1p	p36.12	21877674	21905626	a	0,00095198	-0,77409018
204784_s_at	MLF1	3	3q	q25.32	159771687	159805878	a	0,00096206	-1,60572083
1553602_at	MUCL1	12	12q	q13.2	53534566	53538437	b	4,13E-21	5,26160053
214079_at	DHRS2	14	14q	q11.2	23182093	23184686	b	7,40E-12	6,07114177
223748_at	SLC4A11	20	20p	p13	3156063	3166373	b	7,93E-10	2,22123385
226553_at	TMPRSS2	21	21q	q22.3	41758174	41791989	b	1,23E-09	1,24805783
203021_at	SLPI	20	20q	q13.12	43314292	43316616	b	1,39E-09	3,58791781
201525_at	APOD	3	3q	q29	196776885	196787657	b	1,51E-09	4,13028273
231098_at	---	8	8q	q11.23	54586901	54587381	b	1,84E-08	1,99477218
228064_at	C22orf36	22	22q	q11.23	23311602	23312245	b	8,02E-08	1,3144069
211682_x_at	UGT2B28	4	4q	q13.2	70180805	70195356	b	1,59E-07	6,76107504
226812_at	---	11	11q	q12.1	58103517	58138386	b	2,39E-07	0,21132599
201087_at	PXN	12	12q	q24.31	119132639	119187892	b	3,38E-07	0,75117881
217276_x_at	SERHL2	22	22q	q13.2	41281178	41300005	b	1,06E-06	5,41658163
206463_s_at	DHRS2	14	14q	q11.2	23177396	23184470	b	3,29E-06	5,20892144
200831_s_at	SCD	10	10q	q24.31	102096870	102114573	b	3,87E-06	2,14221968
228523_at	NANOS1	10	10q	q26.11	120779907	120781025	b	4,66E-06	2,39161748
217284_x_at	SERHL2	22	22q	q13.2	41279970	41300005	b	5,14E-06	5,60209707
1561817_at	---	17	17p	p13.1	7901044	7902449	b	5,22E-06	4,32106468
214243_s_at	SERHL	22	22q	q13.2	41279868	41300335	b	5,90E-06	5,36425574
1553394_a_at	TFAP2B	6	6p	p12.3	50894564	50919073	b	5,92E-06	3,50415633
224146_s_at	ABCC11	16	16q	q12.1	46758441	46838806	b	9,46E-06	5,03509997
201931_at	ETFA	15	15q	q24.2	74295690	74390784	b	1,36E-05	1,2069696
206714_at	ALOX15B	17	17p	p13.1	7883126	7893174	b	1,38E-05	6,19591068
235578_at	ABCC9	12	12p	p12.1	21841184	21842104	b	2,08E-05	0,21581809

Table S1: List of the probe sets constituting of the Cowden signature

pbs	gene symbol	chrom	arm	cytogenet	start	end	genes cluster	p-value	fold-change
1555416_a_at	ALOX15B	17	17p	p13.1	7883198	7892608	b	2,13E-05	6,12786595
218764_at	PRKCH	14	14q	q23.1	61086426	61087443	b	2,23E-05	1,14477299
208818_s_at	COMT	22	22q	q11.21	18309332	18336464	b	2,33E-05	1,41951051
208792_s_at	CLU	8	8p	p21.1	27511568	27524745	b	2,61E-05	2,23869544
1553434_at	CYP4Z2P	1	1p	p33	47305708	47345938	b	3,11E-05	3,80978764
222454_s_at	PARVA	11	11p	p15.3	12355677	12508919	b	3,12E-05	0,64856798
204818_at	HSD17B2	16	16q	q23.3	80626363	80689638	b	3,15E-05	3,57509996
217388_s_at	KYNU	2	2q	q22.2	143513697	143516350	b	3,44E-05	3,86989292
219405_at	TRIM68	11	11p	p15.4	4576477	4580220	b	3,78E-05	2,05677153
208817_at	COMT	22	22q	q11.21	18309332	18336464	b	5,49E-05	1,5477042
206165_s_at	CLCA2	1	1p	p22.3	86662412	86694519	b	5,51E-05	4,74079133
208791_at	CLU	8	8p	p21.1	27511568	27524745	b	5,68E-05	2,43233867
204675_at	SRD5A1	5	5p	p15.31	6686562	6722673	b	5,76E-05	3,0060438
210959_s_at	SRD5A1	5	5p	p15.31	6686531	6721746	b	5,81E-05	3,31554203
214461_at	LBP	20	20q	q11.23	36408321	36439074	b	5,91E-05	5,9815393
220615_s_at	MLSTD1	12	12p	p11.22	29193498	29378272	b	6,09E-05	2,21114269
214469_at	HIST1H2AE	6	6p	p22.1	26325126	26325686	b	6,22E-05	2,84197087
209389_x_at	DBI	2	2q	q14.2	119841040	119846448	b	6,43E-05	1,64896627
228888_at	STAC2	17	17q	q12	34620314	34621167	b	6,58E-05	1,66368184
206099_at	PRKCH	14	14q	q23.1	60858410	61086476	b	6,98E-05	1,0625015
206164_at	CLCA2	1	1p	p22.3	86662412	86694519	b	8,28E-05	3,30727816
215603_x_at	GGT1	22	22q	q11.21	19892289	19893772	b	0,00010067	2,62266342
211162_x_at	SCD	10	10q	q24.31	102097816	102110969	b	0,00010098	2,50479786
244127_at	---	15	15q	q26.1	89276819	89279364	b	0,0001026	0,34869137
209919_x_at	GGT1	22	22q	q11.23	23353218	23354942	b	0,00010299	3,31524649
211708_s_at	SCD	10	10q	q24.31	102097074	102110667	b	0,00010944	2,51370783
203180_at	ALDH1A3	15	15q	q26.3	99237583	99274352	b	0,00011604	2,02571243
225342_at	AK3L1	1	1p	p31.3	65467682	65470416	b	0,00011639	1,8757012
208284_x_at	GGT1	22	22q	q11.23	23329182	23354960	b	0,00011974	3,0690712
210663_s_at	KYNU	2	2q	q22.2	143351695	143463574	b	0,00012211	3,8856584
207275_s_at	ACSL1	4	4q	q35.1	185914256	185961696	b	0,00012637	2,3180205
216920_s_at	TARP	7	7p	p14.1	38245704	38365141	b	0,00012719	3,20209176
215806_x_at	TARP	7	7p	p14.1	38245712	38365108	b	0,00012767	3,18158605
217188_s_at	C14orf1	14	14q	q24.3	75187650	75196967	b	0,00013312	0,63643437
211237_s_at	FGFR4	5	5q	q35.2	176449173	176457376	b	0,00013676	1,47467975
211138_s_at	KMO	1	1q	q43	239762272	239822212	b	0,00014245	2,76841587
239108_at	MLSTD1	12	12p	p11.22	29379397	29379815	b	0,00014356	2,22705312
209306_s_at	SWAP70	11	11p	p15.4	9642207	9731077	b	0,00014401	0,7484859
209917_s_at	TP53AP1	7	7q	q21.12	86808774	86812744	b	0,0001453	1,46920938
229616_s_at	GRAMD2	15	15q	q23	70239200	70241200	b	0,0001579	2,78755674
211070_x_at	DBI	2	2q	q14.2	119841054	119846586	b	0,00016042	1,55491548
201951_at	ALCAM	3	3q	q13.11	106568811	106778436	b	0,00016048	1,49099448
211417_x_at	GGT1	22	22q	q11.23	23353221	23354972	b	0,00016112	3,00215762
203060_s_at	PAPSS2	10	10q	q23.2	89409655	89497440	b	0,00016367	2,43170477
205306_x_at	KMO	1	1q	q43	239762056	239825569	b	0,00017164	2,77178311
207131_x_at	GGT1	22	22q	q11.23	23309717	23354935	b	0,00017899	3,30597864
204044_at	QPRT	16	16p	p11.2	29597975	29616457	b	0,00017973	2,2213318
39248_at	AQP3	9	9p	p13.3	33431153	33431590	b	0,00018682	3,72493523
204385_at	KYNU	2	2q	q22.2	143351693	143516344	b	0,00019004	2,79802665
209813_x_at	TARP	7	7p	p14.1	38265768	38323770	b	0,00019443	3,14040823
211382_s_at	TACC2	10	10q	q26.13	123959546	124003841	b	0,00019996	1,11156788
211144_x_at	TARP	7	7p	p14.1	38245720	38337083	b	0,0002095	2,93273965
203914_x_at	HPGD	4	4q	q34.1	175647981	175680194	b	0,00021531	5,28815673
201963_at	ACSL1	4	4q	q35.1	185913748	185961675	b	0,00022004	1,99160722
202428_x_at	DBI	2	2q	q14.2	119841630	119846586	b	0,0002208	1,44687895
211416_x_at	GGTLC1	20	20p	p11.21	23913690	23915432	b	0,00022233	2,3975293
230323_s_at	TMEM45B	11	11q	q24.3	129234154	129234614	b	0,00022364	3,27192547
204941_s_at	ALDH3B2	11	11q	q13.2	67186217	67198649	b	0,00022412	3,37756192
211652_s_at	LBP	20	20q	q11.23	36408333	36438722	b	0,00022698	4,17556242
204942_s_at	ALDH3B2	11	11q	q13.2	67186217	67198641	b	0,00023023	3,18226204
203058_s_at	PAPSS2	10	10q	q23.2	89409627	89497442	b	0,00023408	2,64075798
211056_s_at	SRD5A1	5	5p	p15.31	6686581	6722509	b	0,00023877	2,83569733
203913_s_at	HPGD	4	4q	q34.1	175647784	175680550	b	0,00024678	5,47111179
244044_at	---	6	6q	q23.2	132056790	132057615	b	0,00024776	3,67060454
204615_x_at	IDI1	10	10p	p15.3	1076028	1080113	b	0,00025083	1,98609102
209699_x_at	AKR1C2	10	10p	p15.1	5021964	5036050	b	0,00025286	3,39233773
212249_at	PIK3R1	5	5q	q13.1	67557872	67633403	b	0,00025488	1,63299792

Table S1: List of the probe sets constituting of the Cowden signature

pbs	gene symbol	chrom	arm	cytogenet	start	end	genes cluster	p-value	fold-change
208881_x_at	IDI1	10	10p	p15.3	1075964	1080113	b	0,00025724	2,06918302
201998_at	ST6GAL1	3	3q	q27.3	188239223	188279035	b	0,00026831	1,90259127
201960_s_at	MYCBP2	13	13q	q22.3	76516793	76799178	b	0,00026904	1,25993921
205020_s_at	ARL4A	7	7p	p21.3	12693814	12695328	b	0,00026934	1,54729602
243585_at	ATP13A5	3	3q	q29	194475271	194485471	b	0,00027409	2,97554918
205030_at	FABP7	6	6q	q22.31	123142562	123146915	b	0,00028463	6,11443537
202289_s_at	TACC2	10	10q	q26.13	123913348	124004043	b	0,0002919	0,96151831
206166_s_at	CLCA2	1	1p	p22.3	86662412	86693824	b	0,0002928	3,24137259
209016_s_at	KRT7	12	12q	q13.13	50913304	50928976	b	0,00029778	2,21738045
211548_s_at	HPGD	4	4q	q34.1	175648186	175680194	b	0,00029795	5,29392807
206827_s_at	TRPV6	7	7q	q34	142279082	142279807	b	0,00030178	1,31266832
1554119_at	C16orf57	16	16q	q13	56592823	56604971	b	0,00030434	0,2548574
214598_at	CLDN8	21	21q	q22.11	30508199	30510262	b	0,0003176	4,05687209
201952_at	ALCAM	3	3q	q13.11	106568811	106778436	b	0,00032481	1,48758326
212025_s_at	FLII	17	17p	p11.2	18088857	18102791	b	0,0003418	0,55468059
212240_s_at	PIK3R1	5	5q	q13.1	67557872	67633403	b	0,00034391	1,40115108
240242_at	ATP13A4	3	3q	q29	194599452	194599886	b	0,00037029	3,42295552
206561_s_at	AKR1B10	7	7q	q33	133863164	133876698	b	0,00037114	3,68068094
205073_at	CYP2J2	1	1p	p32.1	60131567	60165011	b	0,00038312	2,31313434
221669_s_at	ACAD8	11	11q	q25	133628674	133640765	b	0,00039825	1,19348241
222938_x_at	ENPP3	6	6q	q23.2	132000136	132109898	b	0,00040018	2,83086577
232732_at	---	12	12q	q13.13	52194071	52197657	b	0,00044817	0,29912238
204058_at	ME1	6	6q	q14.2	83976830	84197506	b	0,00045399	2,35478573
211549_s_at	HPGD	4	4q	q34.1	175649658	175680177	b	0,00045648	4,69523973
215870_s_at	PLA2G5	1	1p	p36.13	20258709	20290246	b	0,00048765	0,98863865
226226_at	TMEM45B	11	11q	q24.3	129227577	129234506	b	0,00049144	2,41878957
219551_at	EAF2	3	3q	q13.33	123036719	123088004	b	0,00050064	2,07334444
224210_s_at	PXMP4	20	20q	q11.22	31758470	31771698	b	0,00050493	1,89948656
211653_x_at	AKR1C2	10	10p	p15.1	5022124	5035968	b	0,00050627	3,05372772
1555037_a_at	IDH1	2	2q	q33.3	208809274	208827155	b	0,00050665	1,27105584
224856_at	FKBP5	6	6p	p21.31	35649344	35651470	b	0,00056818	2,89847913
223098_s_at	LONP2	16	16q	q12.1	46887544	46944908	b	0,00056927	1,48762499
242951_at	TMEM86A	11	11p	p15.1	18683349	18683929	b	0,00057571	1,83141384
1553132_a_at	TC2N	14	14q	q32.12	91318691	91403619	b	0,00057617	1,49682156
1556190_s_at	PRNP	8	8q	q11.23	54590291	54592069	b	0,00057665	2,05429822
212096_s_at	MTUS1	8	8p	p22	17545587	17657279	b	0,00058648	1,04892879
201193_at	IDH1	2	2q	q33.3	208809198	208828051	b	0,00059687	1,23300256
204560_at	FKBP5	6	6p	p21.31	35651422	35764692	b	0,00060224	3,13023312
1554712_a_at	GLYATL2	11	11q	q12.1	58358117	58368575	b	0,00061944	3,79089802
1558846_at	PNLIPRP3	10	10q	q25.3	118177371	118227456	b	0,00062537	4,82738251
228206_at	HS3ST4	16	16p	p12.1	25611857	26056506	b	0,00062726	0,81520891
233651_s_at	MPND	19	19p	p13.3	4294704	4310999	b	0,00063875	0,66564107
203474_at	IQGAP2	5	5q	q13.3	75734904	76039711	b	0,00065723	1,64902198
219428_s_at	PXMP4	20	20q	q11.22	31758228	31771767	b	0,00065965	2,08459945
224840_at	FKBP5	6	6p	p21.31	35649344	35651470	b	0,00066243	2,84432411
204348_s_at	AK3L1	1	1p	p31.3	65386494	65465286	b	0,0007717	1,89655465
244179_x_at	---	22	22q	q11.21	17155043	17161964	b	0,00077266	0,53902253
208690_s_at	PDLIM1	10	10q	q23.33	96987321	97040771	b	0,00079423	1,56036292
219916_s_at	RNF39	6	6p	p21.33	30146025	30151607	b	0,00079695	1,27195124
1566472_s_at	RETSAT	2	2p	p11.2	85393813	85397280	b	0,00080373	1,59032044
1554385_a_at	PADI2	1	1p	p36.13	17278112	17318517	b	0,00081086	1,67926267
242103_at	TMEM86A	11	11p	p15.1	18680192	18680682	b	0,00081262	1,77694717
203059_s_at	PAPSS2	10	10q	q23.2	89409637	89495796	b	0,00081674	1,86105193
226489_at	TMCC3	12	12q	q22	93486473	93533968	b	0,00082776	1,96882684
201301_s_at	ANXA4	2	2p	p14	69822727	69906278	b	0,00085355	0,72378532
1570107_at	---	NA	NA	NA	NA	NA	b	0,00085954	0,37296143
218124_at	RETSAT	2	2p	p11.2	85422732	85430877	b	0,00086604	1,34061456
217738_at	NAMPT	7	7q	q22.2	105675974	105712874	b	0,00086764	2,03993217
230630_at	AK3L1	1	1p	p31.3	65467357	65467807	b	0,00092133	1,56927678
207102_at	AKR1D1	7	7q	q34	137411735	137453590	b	0,00092631	4,25221527
227570_at	TMEM86A	11	11p	p15.1	18680426	18681235	b	0,00093177	1,45260008
238710_at	TMEM86A	11	11p	p15.1	18682425	18683106	b	0,00094224	2,05186176
227490_at	WDFY2	13	13q	q14.3	51056638	51232610	b	0,00094697	0,8035681
202275_at	G6PD	X	Xq	q28	153412799	153428663	b	0,00096482	2,04682335
235256_s_at	GALM	2	2p	p22.1	38810198	38815029	b	0,00097372	1,46814448
202605_at	GUSB	7	7q	q11.21	65063109	65084631	b	0,00097842	2,09457618

Table S3: Detailled immunohistochemistry results with percentage of immunoreactive tumoral cells and intensity of signal. Molecular apocrine status, germline PTEN mutation and stanford classification are also provided

Tumor Sample ID	Molecular apocrine	RNA available	AR %	AR Intensity	ER %	ER Intensity	PR %	PR Intensity	ERBB2 %	ERBB2 Intensity	GCDFP15 %	GCDFP15 Intensity	GGT1 %	GGT1 Intensity	PTEN %	PTEN Intensity	EGFR %	EGFR Intensity	PTEN germline mutation status	Molecular classification (Sorlie)	Molecular classification (Hu)
_003	No	Yes	100	3	15	1	75	3	100	3 X	1	2	0	0	100	2	0	0	WT	Luminal B	Luminal B
_005	No	Yes	90	2	90	2	0	0	0	0	0	0	0	0	90	2	0	0	WT	Luminal A	Luminal A
_008	No	Yes	0	0	0	0	0	0	0	0	0	0	0	0	100	2	20	1	WT	Basal-like	Basal-like
_009	No	Yes	90	2	90	2	100	2	0	0	0	0	0	0	0	0	0	0	WT	Luminal A	Luminal A
_011	No	Yes	90	3	90	2	90	3	0	0	0	0	0	0	0	0	0	0	WT	Luminal A	Luminal B
_013	No	Yes	100	3	80	2	90	3	0	0	40	2	0	0	100	3	0	0	WT	HER2	Luminal B
_014	No	Yes	90	2	na	na	90	3	0	0	25	1	0	0	100	2	0	0	WT	Luminal A	Luminal A
_015	No	Yes	0	0	0	0	0	0	0	0	0	0	0	0	0	0	66	2	WT	Basal-like	Basal-like
_016	No	Yes	90	2	100	3	90	3	0	0	0	0	0	0	100	3	0	0	WT	Luminal A	Luminal A
_017	No	Yes	100	3	100	3	50	2	0	0	0	0	0	0	100	2	0	0	WT	Luminal A	Luminal A
_018	No	Yes	90	3	25	2	0	0	0	0	0	0	0	0	100	2	0	0	WT	HER2	Normal-like
_020	No	Yes	0	0	0	0	0	0	0	0	0	0	0	0	66	1	0	0	WT	Basal-like	Basal-like
_022	No	Yes	100	3	100	3	50	2	0	0	0	0	0	0	100	2	0	0	WT	Luminal A	Luminal A
_024	No	Yes	90	3	90	2	20	1	0	0	0	0	0	0	100	2	0	0	WT	Luminal A	Luminal A
_025	No	Yes	100	3	90	2	25	2	0	0	0	0	0	0	100	2	0	0	WT	Luminal B	Luminal B
_028	No	Yes	90	3	100	3	50	2	0	0	0	0	0	0	100	2	0	0	WT	Luminal A	Luminal B
_030	No	Yes	na	na	na	na	na	na	na	na	na	na	na	na	na	na	na	na	WT	Luminal A	HER2
_032	No	Yes	90	3	80	2	90	3	0	0	1	1	0	0	100	2	0	0	WT	Normal-like	HER2
_033	No	Yes	15	2	90	2	5	1	0	0	5	2	0	0	100	1	0	0	WT	Luminal A	Luminal A
_034	No	Yes	90	2	100	3	30	3	0	0	0	0	0	0	100	1	0	0	WT	Basal-like	Basal-like
_035	No	Yes	30	2	90	2	10	2	0	0	0	0	0	0	90	2	0	0	WT	HER2	Luminal B
_038	No	Yes	90	3	100	3	0	0	0	0	5	1	0	0	100	2	0	0	WT	HER2	Luminal B
_039	No	Yes	100	3	100	3	90	3	80	3 X	0	0	0	0	100	2	0	0	WT	Normal-like	Normal-like
_040	No	Yes	40	2	30	2	90	2	0	0	0	0	0	0	40	1	0	0	WT	Luminal B	HER2
_041	No	Yes	0	0	80	2	30	2	0	0	5	2	0	0	90	2	0	0	WT	Luminal A	Luminal A
_046	Yes	Yes	100	3	0	0	0	0	0	0	30	2	90	3	100	2	70	2	WT	HER2	Normal-like
_047	No	Yes	90	2	90	2	0	0	0	0	0	0	0	0	90	1	0	0	WT	Luminal B	Luminal B
_048	No	Yes	50	2	75	2	90	2	0	0	0	0	0	0	100	2	0	0	WT	Luminal A	Luminal A
_049	No	Yes	90	3	90	3	90	2	0	0	20	2	0	0	100	2	0	0	WT	Normal-like	Normal-like
_051	No	Yes	0	0	0	0	0	0	0	0	0	0	0	0	na	na	0	0	WT	Basal-like	Basal-like
_052	No	Yes	100	3	70	2	10	2	0	0	0	0	0	0	100	3	0	0	WT	Luminal B	Luminal B
_054	No	Yes	33	3	90	3	1	1	0	0	0	0	0	0	100	2	0	0	WT	Luminal A	HER2
_060	No	Yes	0	0	0	0	0	0	0	0	0	0	0	0	100	1	40	2	WT	Basal-like	Basal-like
_061	No	Yes	80	2	100	3	90	2	0	0	1	2	0	0	100	2	0	0	WT	Normal-like	Luminal A
_062	No	Yes	15	2	80	2	1	1	0	0	0	0	0	0	100	1	0	0	WT	Luminal A	HER2
_063	No	Yes	90	3	75	2	90	2	0	0	0	0	0	0	100	2	0	0	WT	Luminal A	Luminal A

Table S3: Detailled immunohistochemistry results with percentage of immunoreactive tumoral cells and intensity of signal. Molecular apocrine status, germline PTEN mutation and stanford classification are also provided

Tumor Sample ID	Molecular apocrine	RNA available	AR %	AR Intensity	ER %	ER Intensity	PR %	PR Intensity	ERBB2 %	ERBB2 Intensity	GCDFP15 %	GCDFP15 Intensity	GGT1 %	GGT1 Intensity	PTEN %	PTEN Intensity	EGFR %	EGFR Intensity	PTEN germline mutation status	Molecular classification (Sorlie)	Molecular classification (Hu)
_065	No	Yes	5	2	0	0	0	0	0	0	0	0	0	0	100	1	40	1	WT	Basal-like	Basal-like
_066	No	Yes	100	3	100	3	80	3	0	0	30	2	0	0	100	2	0	0	WT	Luminal A	Luminal A
_067	No	Yes	0	0	0	0	0	0	0	0	0	0	0	0	100	1	100	3	WT	Basal-like	Basal-like
_068	No	Yes	100	3	75	2	5	2	0	0	1	2	0	0	100	2	0	0	WT	Normal-like	Normal-like
_070	No	Yes	0	0	20	2	0	0	0	0	0	0	0	0	75	1	0	0	WT	Luminal B	Luminal A
_071	No	Yes	80	3	60	2	80	3	100	3 X	0	0	0	0	100	3	0	0	WT	HER2	Normal-like
_072	No	Yes	90	3	90	2	25	2	0	0	0	0	0	0	80	1	0	0	WT	HER2	Normal-like
_074	No	Yes	100	3	20	2	0	0	0	0	0	0	0	0	100	3	0	0	WT	Normal-like	Normal-like
_075	No	Yes	90	3	2	2	0	0	100	3 X	0	0	0	0	100	2	0	0	WT	HER2	HER2
_077	No	Yes	100	3	80	2	90	3	0	0	1	2	0	0	100	2	0	0	WT	Luminal A	Luminal A
_078	No	Yes	100	3	90	1	0	0	0	0	0	0	0	0	100	2	0	0	WT	Luminal A	Luminal B
_079	No	Yes	90	2	90	2	66	2	0	0	0	0	0	0	100	2	0	0	WT	Luminal B	Luminal B
_080	No	Yes	na	na	na	na	na	na	na	na	na	na	na	na	na	na	na	na	WT	Luminal B	Luminal B
_081	No	Yes	90	3	25	2	90	2	0	0	0	0	0	0	90	1	0	0	WT	Luminal B	Luminal A
_082	No	Yes	100	3	80	2	60	2	0	0	0	0	0	0	100	1	0	0	WT	Luminal A	HER2
_084	No	Yes	75	3	90	2	40	2	25	2 X	0	0	0	0	100	2	0	0	WT	Luminal B	Luminal B
_085	No	Yes	20	2	20	1	75	3	0	0	0	0	0	0	90	1	0	0	WT	Luminal A	Luminal A
_086	No	Yes	40	2	90	3	15	2	0	0	0	0	0	0	100	2	0	0	WT	Luminal B	Luminal B
_087	No	Yes	40	3	80	1	1	2	0	0	0	0	0	0	100	2	0	0	WT	Luminal B	Luminal B
_088	No	Yes	90	2	90	2	10	2	0	0	0	0	0	0	90	2	0	0	WT	Normal-like	Normal-like
_089	No	Yes	100	3	90	2	80	3	0	0	0	0	90	2	95	2	0	0	WT	Luminal A	Luminal A
_090	No	Yes	75	3	66	2	80	2	0	0	0	0	0	0	100	2	0	0	WT	Normal-like	HER2
_092	No	Yes	0	0	0	0	0	0	0	0	0	0	0	0	100	2	0	0	WT	Basal-like	Basal-like
_095	No	Yes	100	3	100	3	1	1	0	0	0	0	0	0	90	2	0	0	WT	Luminal A	Normal-like
_096	Yes	Yes	100	3	0	0	0	0	100	3 X	50	2	60	2	100	2	10	1	WT	HER2	HER2
_097	No	Yes	75	3	90	2	100	3	0	0	0	0	0	0	100	2	0	0	WT	Luminal A	Luminal A
_099	No	Yes	100	3	90	3	66	3	0	0	30	2	0	0	100	2	0	0	WT	Luminal A	Luminal A
_100	No	Yes	0	0	0	0	0	0	0	0	0	0	0	0	100	2	0	0	WT	Basal-like	Basal-like
_104	No	Yes	0	0	0	0	0	0	0	0	0	0	0	0	90	1	0	0	WT	Basal-like	Basal-like
_105	No	Yes	90	2	100	3	90	3	0	0	0	0	0	0	80	1	0	0	WT	Normal-like	Luminal A
_106	No	Yes	40	1	100	3	20	2	0	0	0	0	0	0	100	1	0	0	WT	Luminal B	Luminal B
_107	No	Yes	50	2	100	3	30	2	0	0	0	0	0	0	100	2	0	0	WT	Luminal A	Luminal A
_108	No	Yes	90	3	15	2	25	2	100	3 X	0	0	0	0	100	2	0	0	WT	HER2	HER2
_110	No	Yes	80	3	100	3	5	2	0	0	1	2	0	0	100	2	0	0	WT	HER2	Luminal B
_111	No	Yes	100	3	100	3	30	2	0	0	0	0	0	0	100	3	0	0	WT	Luminal A	Luminal A
_113	Yes	Yes	100	3	0	0	0	0	0	0	100	2	100	3	0	0	40	2	c.209+5G>A	Normal-like	Normal-like

Table S3: Detailled immunohistochemistry results with percentage of immunoreactive tumoral cells and intensity of signal. Molecular apocrine status, germline PTEN mutation and stanford classification are also provided

Tumor Sample ID	Molecular apocrine	RNA available	AR %	AR Intensity	ER %	ER Intensity	PR %	PR Intensity	ERBB2 %	ERBB2 Intensity	GCDFP15 %	GCDFP15 Intensity	GGT1 %	GGT1 Intensity	PTEN %	PTEN Intensity	EGFR %	EGFR Intensity	PTEN germline mutation status	Molecular classification (Sorlie)	Molecular classification (Hu)
_116	Yes	Yes	100	3	100	3	100	3	0	0	0	0	40	2	0	0	0	0	c.1007dupA p.Tyr336X	Normal-like	Normal-like
_118	Yes	Yes	100	3	0	0	0	0	0	0	100	2	100	3	0	0	80	3	c.209+5G>A	Normal-like	Normal-like
_S243	na	No	80	2	100	3	100	3	0	0	0	0	5	1	0	0	0	0	c.323T>C p.Leu108Pro	na	na
_S295	na	No	100	3	0	0	0	0	0	0	100	3	80	2	0	0	na	na	c.209+5G>A	na	na
_S362	na	No	100	3	70	2	60	3	0	0	0	0	10	1	0	0	0	0	c.69dupA p.Asp24ArgfsX20	na	na
_S403	na	No	80	2	100	2	100	3	0	0	0	0	0	0	0	0	0	0	c.801+1delG	na	na
_S574	na	No	100	3	100	3	100	3	0	0	0	0	5	1	0	0	0	0	c.491delA p.Lys164ArgfsX3	na	na
_S681	na	No	60	2	80	3	10	3	30	1 X	10	3	5	1	0	0	0	0	c.830C>G p.Thr277Arg	na	na
_S712	na	No	100	3	100	3	0	0	na	0	0	0	10	1	0	0	na	na	c.592delA p.Met198X	na	na
_S730	na	No	80	2	100	3	90	3	20	1 X	40	2	0	0	0	0	0	0	c.493G>A p.Gly165Arg	na	na
_S732	na	No	80	3	80	2	80	3	0	0	10	2	5	1	5	1	0	0	c.510T>G p.Ser170Arg	na	na
_S89	na	No	80	3	100	3	100	3	0	0	0	0	0	0	0	0	na	na	c.158_159insATAC p.Val54TyrfsX10	na	na
_S891	na	No	100	3	0	0	0	0	80	2 X	100	3	100	2	20	1	0	0	c.209+5G>A	na	na
_S912	na	No	100	3	100	3	60	2	0	0	5	1	10	1	0	0	0	0	c.632_633delGC p.Cys211X	na	na

Table S4: Immunohistochemistry scoring for the three groups of tumors

antibody	score	Cowden (n=15)	Molec. Apo. (n=5)	Others (n=69)
AR	0	0	0	11
	1	0	0	1
	2	15 (100%)	5 (100%)	55 (80%)
	na	0	0	2
ER	0	4	4	10
	1	0	0	1
	2	11 (73%)	1 (20%)	55 (80%)
	na	0	0	3
PR	0	5	4	18
	1	1	0	9
	2	9 (60%)	1 (20%)	40 (58%)
	na	0	0	2
ERBB2	0	11	4	61
	+	2	0	0
	++	1	0	1
	+++	0 (0%)	1 (20%)	5 (7%)
	na	1	0	2
GCDFP15	0	7	1	53
	1	3	0	9
	2	5 (33%)	4 (80%)	5 (7%)
	na	0	0	2
GGT1	0	3	0	66
	1	7	0	0
	2	5 (33%)	5 (100%)	1 (1,5%)
	na	0	0	2
PTEN	0	13	3	3
	1	1	0	0
	2	1 (7%)	2 (40%)	63 (91%)
	na	0	0	3
EGFR	0	10	1	62
	1	0	1	0
	2	2 (13%)	3 (60%)	5 (7%)
	na	3	0	2

Table S5 : ArrayCGH derived rate of perturbation and percentage of tumoral cells for the five Cowden and non Cowden apocrine carcinomas

Tumor Sample ID	Rate of perturbation	Percentage of tumor cells
_046	0,27	90 %
_096	0,43	85 %
_113	0,08	90 %
_116	0,05	95 %
_118	0,12	75 %

Table S6: List of probe sets distinguishing between Cowden and non-Cowden tumors within apocrine breast cancers

pbs	Gene Symbol	chrom	arm	cytogenet	start	end	genes Cluster	p-value	fold change
219308_s_at	AK5	1	1p	p31.1	77721586	77798240	a	8,78E-05	2,97241088
209137_s_at	USP10	16	16q	q24.1	83291084	83370670	a	0,00050589	0,34589039
201200_at	CREG1	1	1q	q24.2	165776880	165789628	a	0,00066233	0,83794043
213032_at	NFIB	9	9p	p23	14071847	14073499	a	0,00100488	1,66511871
203698_s_at	FRZB	2	2q	q32.1	183407530	183439729	a	0,00103472	4,23800028
1553971_a_at	GATS	7	7q	q22.1	99645222	99649244	a	0,00148594	0,9206392
202959_at	MUT	6	6p	p12.3	49506031	49538967	a	0,00159112	0,88675453
238860_at	C6orf130	6	6p	p21.1	41142031	41142703	a	0,00165907	1,11116848
206799_at	SCGB1D2	11	11q	q12.3	61766301	61768855	a	0,00178056	7,25805534
204863_s_at	IL6ST	5	5q	q11.2	55271508	55326529	a	0,00180232	1,34424801
225637_at	DEF8	16	16q	q24.3	88555981	88561968	a	0,00215187	1,0290585
1558103_a_at	NA	15	15q	q25.2	81593881	81596691	a	0,00243804	1,48693516
244581_at	ZBTB20	3	3q	q13.31	115859877	115860393	a	0,00245561	0,20364107
215667_x_at	NA	7	7q	q11.23	72114615	72157707	a	0,00248963	0,36169586
236042_at	NA	12	12p	p13.33	954456	955373	a	0,00290093	0,45275917
227642_at	TFCP2L1	2	2q	q14.2	121690635	121691154	a	0,00293946	3,03784325
201939_at	PLK2	5	5q	q11.2	57785570	57791844	a	0,0029999	1,14440281
244740_at	MGC9913	19	19q	q13.43	61681330	61697525	a	0,00306356	1,29502017
220432_s_at	CYP39A1	6	6p	p12.3	46625492	46728322	a	0,00320576	2,18643041
217800_s_at	NDFIP1	5	5q	q31.3	141468619	141512523	a	0,00323594	0,9943486
225108_at	AGPS	2	2q	q31.2	178111252	178116808	a	0,00337567	1,22247908
213260_at	FOXC1	6	6p	p25.3	1555249	1559126	a	0,0035713	1,19588428
221530_s_at	BHLHB3	12	12p	p12.1	26164065	26169271	a	0,0036432	2,28680449
203263_s_at	ARHGEF9	X	Xq	q11.1	62771572	62891718	a	0,00378353	0,86814785
204421_s_at	FGF2	4	4q	q27	123967435	124036069	a	0,00382975	1,27477502
218634_at	PHLDA3	1	1q	q32.1	199701245	199704856	a	0,00385296	0,63092334
223495_at	CCDC8	19	19q	q13.32	51605425	51608681	a	0,00390874	1,432545
228584_at	SGCB	4	4q	q12	52582888	52584036	a	0,0039775	1,55846189
235207_at	NA	17	17q	q12	30919254	30925515	a	0,00436341	1,43876108
204917_s_at	MLLT3	9	9p	p21.3	20334967	20612451	a	0,00456504	1,526166
211000_s_at	IL6ST	5	5q	q11.2	55279145	55307879	a	0,00456988	1,40256905
237223_at	NA	2	2p	p21	47406500	47406939	a	0,00469942	0,17889164
211429_s_at	SERPINA1	15	15q	q22.31	63855585	63856741	a	0,00471544	2,94924474
227882_at	FKRP	19	19q	q13.32	51953037	51953676	a	0,00499437	0,66126378
244261_at	IL28RA	1	1p	p36.11	24353233	24353698	a	0,00501423	0,98754073
202925_s_at	PLAGL2	20	20q	q11.21	30243967	30259192	a	0,00512747	0,40843336
226235_at	LOC339290	18	18p	p11.31	5227888	5229389	a	0,00522715	0,61848359
210073_at	ST8SIA1	12	12p	p12.1	22245240	22378500	a	0,00584265	2,2486084
215440_s_at	BEX4	X	Xq	q22.1	102356736	102358666	a	0,00586605	1,34683303
221796_at	NTRK2	9	9q	q21.33	86616703	86620437	a	0,00606784	5,061113
226142_at	GLIPR1	12	12q	q21.2	74179310	74181975	a	0,00635552	0,95621059
202965_s_at	CAPN6	X	Xq	q22.3	110374986	110400407	a	0,00643439	2,79622302
242053_at	NA	2	2q	q11.2	99131158	99137531	a	0,00644198	1,15600309
203636_at	MID1	X	Xp	p22.2	10373595	10761687	a	0,00656577	1,16659136
225810_at	MTMR10	15	15q	q13.3	29018435	29020286	a	0,00714176	0,96408267
229302_at	TMEM178	2	2p	p22.1	39798071	39798605	a	0,00778917	2,38967329
202794_at	INPP1	2	2q	q32.2	190916683	190944552	a	0,00780384	1,13436123
228345_at	CHIC1	X	Xq	q13.2	72822464	72823669	a	0,0079775	1,98642505
210482_x_at	MAP2K5	15	15q	q23	65622666	65886280	a	0,00800643	0,53102112
210976_s_at	PFKM	12	12q	q13.11	46799392	46826154	a	0,00802032	1,41563579
243996_at	NA	9	9p	p13.3	35776642	35780424	a	0,00807603	0,44533472
212956_at	TBC1D9	4	4q	q31.21	141761385	141811532	a	0,00825642	2,00060466
213158_at	NA	3	3q	q13.31	115516042	115518086	a	0,0083635	1,86632432
204422_s_at	FGF2	4	4q	q27	123967312	124038826	a	0,00842587	2,90617706
212403_at	UBE3B	12	12q	q24.11	108399821	108458890	a	0,00860697	0,77354213
240344_x_at	LYRM7	NA	NA	NA	NA	NA	a	0,00910822	0,86018746
204855_at	SERPINB5	18	18q	q21.33	59295198	59323297	a	0,00911084	3,10957093
221476_s_at	RPL15	3	3p	p24.2	23934341	23937334	a	0,00924368	0,51768127
228131_at	ERCC1	19	19q	q13.32	50602431	50603156	a	0,00932085	1,11691882
208914_at	GGA2	16	16p	p12.1	23382871	23429299	a	0,00949516	0,58254247
209692_at	EYA2	20	20q	q13.12	45052054	45250897	a	0,00956756	1,05363796
213156_at	NA	3	3q	q13.31	115516042	115518086	a	0,00994607	2,00736842
213098_at	RQCD1	2	2q	q35	219141633	219168102	b	4,56E-05	-0,44984361
226216_at	INSR	19	19p	p13.2	7064325	7067184	b	0,00032604	-1,07151451
214444_s_at	PVR	19	19q	q13.31	49838993	49857246	b	0,00034699	-0,50600511
223483_at	SERGEF	11	11p	p15.1	17766174	17991159	b	0,00037356	-0,35479183
201629_s_at	ACP1	2	2p	p25.3	254186	268283	b	0,00040406	-1,01278918
221608_at	WNT6	2	2q	q35	219432789	219447199	b	0,00042051	-0,33083419
203113_s_at	EEF1D	8	8q	q24.3	144733040	144750719	b	0,00042643	-0,32864007

Table S6: List of probe sets distinguishing between Cowden and non-Cowden tumors within apocrine breast cancers

pbs	Gene Symbol	chrom	arm	cytogenet	start	end	genes Cluster	p-value	fold change
235192_at	TP53RK	20	20q	q13.12	44746411	44747644	b	0,00047196	-0,53392691
232475_at	C15orf42	15	15q	q26.1	87969295	87975290	b	0,00050609	-0,515272
217834_s_at	SYNCRIP	6	6q	q14.3	86379008	86409428	b	0,00056425	-0,72049048
231589_at	NA	X	Xq	q28	148849721	148850161	b	0,00060953	-0,45005084
229346_at	NES	1	1q	q23.1	154908828	154909373	b	0,00063362	-0,38651679
1559092_at	SLC9A5	16	16q	q22.1	65852511	65855188	b	0,00067921	-0,44399588
207959_s_at	DNAH9	17	17p	p12	11726948	11813788	b	0,00068912	-0,29216246
222089_s_at	C16orf71	16	16p	p13.3	4738723	4739398	b	0,00098386	-0,59058507
240376_s_at	NA	16	16p	p13.11	16323102	16323721	b	0,00098408	-0,47874976
234708_at	SMUG1	12	12q	q13.13	52861507	52864010	b	0,00111331	-0,26639445
239539_at	NA	13	13q	q14.3	51617631	51618090	b	0,00127168	-0,25016303
204128_s_at	RFC3	13	13q	q13.2	33290306	33308829	b	0,00148569	-1,37701996
229230_at	OSTalpha	3	3q	q29	197438313	197444923	b	0,00150475	-3,7066826
230840_at	LOC388588	1	1p	p36.32	3679249	3682405	b	0,00176449	-0,94255949
1556983_a_at	NA	11	11p	p15.4	9721649	9722007	b	0,00176907	-0,82931521
1561085_at	LOC153910	6	6q	q24.1	142888840	143000719	b	0,00183189	-0,22842432
204913_s_at	SOX11	2	2p	p25.2	5750229	5758967	b	0,0021967	-4,36464987
225439_at	NUDCD1	8	8q	q23.1	110322324	110415494	b	0,002252	-1,11901551
1569303_s_at	RGS20	NA	NA	NA	NA	NA	b	0,00226867	-0,5428651
237527_at	NA	11	11q	q12.2	60236781	60237243	b	0,00235786	-0,50397817
220147_s_at	FAM60A	12	12p	p11.21	31324784	31370340	b	0,00237967	-0,42724916
1566177_at	NA	13	13q	q32.3	99179196	99181299	b	0,00241533	-0,45568627
231936_at	HOXC9	12	12q	q13.13	52680179	52683385	b	0,00243539	-1,6914906
240940_at	NA	2	2q	q31.1	177220628	177221366	b	0,00244055	-0,35296914
212461_at	AZIN1	8	8q	q22.3	103907711	103909075	b	0,00260167	-0,65094362
235573_at	NA	13	13q	q12.3	30607117	30608175	b	0,00263827	-0,95583081
243414_at	PPIL2	22	22q	q11.21	20378253	20378658	b	0,00266058	-0,49752303
210499_s_at	PQBP1	X	Xp	p11.23	48640736	48645305	b	0,00270475	-0,36714418
222818_at	OSBPL10	3	3p	p23	31677321	31998366	b	0,00279309	-0,77622108
206888_s_at	ARHGDIG	16	16p	p13.3	270627	272900	b	0,00280456	-0,23292742
213310_at	EIF2C2	8	8q	q24.3	141601643	141605441	b	0,0028139	-2,17829971
204512_at	HIVEP1	6	6p	p24.1	12120556	12273214	b	0,00286493	-1,52271255
207162_s_at	CACNA1B	9	9q	q34.3	139892061	140136452	b	0,00287142	-0,61955716
1553723_at	GPR97	16	16q	q13	56259672	56280125	b	0,00291934	-0,27400527
240491_at	NA	7	7p	p15.1	29213607	29214054	b	0,00294713	-0,36653522
231687_at	NA	1	1p	p31.1	74815460	74815925	b	0,00306357	-0,47760418
223419_at	FBXW9	19	19p	p13.13	12660730	12668432	b	0,00308141	-0,63525053
218092_s_at	HRB	2	2q	q36.3	228045138	228128082	b	0,00310899	-1,56999225
1570342_at	NKTR	3	3p	p22.1	42617238	42636812	b	0,00345168	-0,37616426
1553840_a_at	CCDC149	4	4p	p15.2	24437678	24590924	b	0,00345837	-0,40962131
203744_at	HMGB3	X	Xq	q28	149902426	149907992	b	0,00347108	-2,04562983
210560_at	GBX2	2	2q	q37.2	236739206	236741391	b	0,00378131	-0,32390925
1553691_at	B3GALNT2	1	1q	q42.3	233679266	233734507	b	0,0038582	-0,51291163
241876_at	MDM4	1	1q	q32.1	202850935	202852311	b	0,00391302	-0,55914909
1552736_a_at	NETO1	18	18q	q22.3	68565766	68686164	b	0,00394071	-0,59346961
201451_x_at	RHEB	7	7q	q36.1	150794727	150847924	b	0,00394533	-0,43975162
219510_at	POLQ	3	3q	q13.33	122632970	122692396	b	0,00405628	-1,08913096
239179_at	NA	10	10q	q22.2	75265243	75265716	b	0,00408584	-1,12295745
1562403_a_at	SLC8A3	14	14q	q24.2	69580724	69616650	b	0,00416556	-0,35308825
1559787_at	NA	2	2p	p14	64926767	64930187	b	0,00418797	-0,3194963
1553984_s_at	NA	2	2q	q37.3	242263834	242265760	b	0,00438209	-0,86973034
1560784_x_at	NA	12	12q	q24.33	131369472	131371264	b	0,00443567	-0,65569192
233567_at	NAV1	1	1q	q32.1	200047252	200056504	b	0,0044453	-0,42558479
220928_s_at	PRDM16	1	1p	p36.32	2975620	3340717	b	0,00447059	-0,38138801
215394_at	PIK3C3	18	18q	q12.3	37871692	37885274	b	0,00450673	-0,438797
1561332_at	ATP13A5	3	3q	q29	194513185	194524492	b	0,00461905	-0,58349056
211705_s_at	SORBS1	10	10q	q23.33	97064771	97190909	b	0,00476666	-0,51774613
210841_s_at	NRP2	2	2q	q33.3	206256259	206316666	b	0,00484906	-0,4442698
208054_at	HERC4	10	10q	q21.3	69383965	69386626	b	0,0048869	-0,3171776
1559800_a_at	NA	16	16p	p13.3	4624108	4630625	b	0,00488961	-0,24731821
1560290_at	NA	16	16p	p13.2	8863375	8864534	b	0,00490228	-0,9147265
215435_at	NA	8	8q	q24.21	131224839	131226981	b	0,0049768	-1,1095143
207332_s_at	TFRC	3	3q	q29	197260746	197293338	b	0,00501539	-0,73696214
231525_at	LOC389124	3	3p	p21.1	51882777	51883232	b	0,00513094	-0,40512215
211184_s_at	USH1C	11	11p	p15.1	17472019	17511633	b	0,00515382	-0,40255086
215760_s_at	SBNO2	19	19p	p13.3	1059218	1083133	b	0,00517514	-0,83214792
236967_at	LOC645249	7	7q	q36.3	156496360	156501881	b	0,00534856	-0,73001249
242514_at	NA	7	7p	p22.2	2770421	2770748	b	0,00536994	-0,9837575
212470_at	SPAG9	17	17q	q21.33	46397044	46479270	b	0,00543207	-0,6608434

Table S6: List of probe sets distinguishing between Cowden and non-Cowden tumors within apocrine breast cancers

pbs	Gene Symbol	chrom	arm	cytogenet	start	end	genes Cluster	p-value	fold change
232930_at	DOCK1	10	10q	q26.2	129094325	129097143	b	0,00566534	-0,81924093
214027_x_at	NA	2	2q	q35	219999367	219999698	b	0,00571602	-0,31869077
239869_at	NA	4	4q	q31.3	152548751	152549249	b	0,00579693	-0,39077239
209176_at	SEC23IP	10	10q	q26.11	121642204	121691647	b	0,00579793	-0,53691792
213863_s_at	OAZ3	1	1q	q21.3	150000551	150002465	b	0,00622063	-0,19056211
234446_at	NA	20	20p	p11.21	23445520	23446706	b	0,00629464	-0,58892403
223489_x_at	EXOSC3	9	9p	p13.2	37770449	37775066	b	0,00633841	-0,55306543
1559870_at	NA	8	8p	p23.1	11240667	11242409	b	0,00651569	-0,639298
200853_at	H2AFZ	4	4q	q23	101088265	101090454	b	0,00652622	-0,99334892
236324_at	MBP	18	18q	q23	72826574	72827061	b	0,00652664	-0,2463503
232352_at	ISL2	15	15q	q24.3	74416185	74421870	b	0,00669765	-0,8472071
205592_at	SLC4A1	17	17q	q21.31	39681283	39701028	b	0,0067176	-0,47368766
218663_at	NCAPG	4	4p	p15.32	17421702	17454229	b	0,00673926	-1,61497446
204735_at	PDE4A	19	19p	p13.2	10424636	10441306	b	0,00675291	-1,00270479
1556613_s_at	LOC203107	8	8q	q22.1	95801319	95826487	b	0,00687514	-0,66444468
1553780_at	MGC23270	14	14q	q32.33	104358582	104361100	b	0,00688853	-0,38459905
230103_at	C10orf72	10	10q	q11.22	49926528	49986047	b	0,00694041	-0,73170178
1556582_at	NA	19	19q	q13.31	49858919	49861269	b	0,00703058	-0,25839451
234397_at	NA	19	19p	p13.12	15793802	15795597	b	0,00703998	-0,55974705
217616_at	NA	21	21q	q22.3	43666688	43667397	b	0,00704661	-0,36758602
232513_x_at	C20orf107	20	20q	q13.31	54541774	54544981	b	0,00710586	-0,42827476
213114_at	NA	1	1p	p36.32	2313178	2325048	b	0,00723974	-0,83853664
230514_s_at	NA	10	10p	p11.23	29618039	29640164	b	0,00725293	-0,27752799
207235_s_at	GRM5	11	11q	q14.2	87880625	88420838	b	0,0075173	-0,38747964
1563008_at	NA	1	1p	p36.31	5690827	5694812	b	0,00752366	-0,43480856
229296_at	NA	9	9q	q34.11	131138978	131685172	b	0,00766077	-1,44325813
1553470_at	DNAH17	17	17q	q25.3	74051431	74078999	b	0,0077033	-0,31367041
1562449_s_at	NA	22	22q	q12.3	32451595	32476714	b	0,00775419	-0,23040838
224441_s_at	USP45	6	6q	q16.3	100036858	100070029	b	0,00778442	-0,55069276
225033_at	LOC286167	8	8q	q24.22	134536273	134539258	b	0,00780259	-1,39379635
239662_x_at	NA	3	3q	q21.3	131094676	131095108	b	0,00781809	-0,43782532
208250_s_at	DMBT1	10	10q	q26.13	124310170	124393242	b	0,00782704	-0,50966232
1554589_at	LENG9	19	19q	q13.42	59664792	59666775	b	0,00783711	-0,63568439
1560638_a_at	NA	NA	NA	NA	NA	NA	b	0,00793425	-0,39048327
234672_s_at	TMEM48	1	1p	p32.3	54003720	54076696	b	0,00793741	-0,45163576
215885_at	SSX2	X	Xp	p11.22	52797047	52806764	b	0,00797876	-0,44761065
210854_x_at	SLC6A8	X	Xq	q28	152610879	152614043	b	0,00827044	-1,46879265
1555586_at	FAM71B	5	5q	q33.3	156524653	156525833	b	0,00833588	-0,41659023
1564031_a_at	RELL2	5	5q	q31.3	140996700	141000814	b	0,00835644	-0,69475026
211025_x_at	COX5B	2	2q	q11.2	97628952	97631088	b	0,00858061	-0,34434071
238080_at	B4GALNT4	11	11p	p15.5	366913	369523	b	0,00858902	-0,66921661
216315_x_at	NA	X	Xq	q21.31	89431190	89431601	b	0,00865522	-0,67575386
1554212_s_at	KCNS2	8	8q	q22.2	99508425	99512197	b	0,00873653	-0,38969002
225600_at	LOC286144	8	8q	q22.1	93964933	94047514	b	0,0088504	-2,12782759
200812_at	CCT7	2	2p	p13.2	73314958	73333640	b	0,00887125	-0,59307311
230208_at	NA	15	15q	q24.1	71399252	71399895	b	0,00894929	-0,31870757
231276_at	PDE3B	11	11p	p15.2	14622373	14622884	b	0,00899741	-0,42804756
1554752_a_at	NA	5	5q	q35.2	172287188	172288713	b	0,00910209	-0,66494188
232460_at	NA	17	17q	q25.3	77278759	77280735	b	0,00911996	-0,53501609
1556923_at	NA	17	17q	q25.1	71481834	71484058	b	0,00912575	-0,4972191
217534_at	FAM49B	8	8q	q24.21	131051865	131052461	b	0,00921489	-1,28165101
233806_at	NA	20	20p	p11.21	23265114	23266791	b	0,00932933	-0,21089716
218420_s_at	C13orf23	13	13q	q13.3	38483280	38496683	b	0,00936809	-0,79400835
241445_at	NA	2	2q	q13	112552740	112553251	b	0,00944731	-1,32098432
214406_s_at	SLC7A4	22	22q	q11.21	19713007	19716146	b	0,00945235	-0,483238
207061_at	ERN1	17	17q	q23.3	59474491	59561208	b	0,00953633	-0,47399557
215026_x_at	SCNN1A	12	12p	p13.31	6353897	6354947	b	0,00954133	-0,62012139
211080_s_at	NEK2	1	1q	q32.3	209911195	209914319	b	0,00968786	-1,32430905
229183_at	KCTD1	18	18q	q11.2	22381041	22381534	b	0,00974498	-0,42420883
210454_s_at	KCNJ6	21	21q	q22.13	37918740	38210241	b	0,00994831	-0,35304046
204702_s_at	NFE2L3	7	7p	p15.2	26159033	26192407	b	0,00995559	-1,89635617
237042_at	NA	6	6q	q15	90687820	90688200	b	0,00998076	-0,4648488

Table S7: Gene ontology and KEGG pathway database interrogation by over expressed genes in Cowden and non Cowden apocrine breast cancers.

Source	Genes cluster over expressed in CD apocrine carcinoma	p-value	Genes cluster over expressed in non CD apocrine carcinoma	p-value
**Gene ontology interrogation**	LIPID_BIOSYNTHETIC_PROCESS	0,000100105	SUBSTRATE_SPECIFIC_ TRANSMEMBRANE_ TRANSPORTER_ACTIVITY	0,001880852
	BIOSYNTHETIC_PROCESS	0,000999927	3__5__CYCLIC_NUCLEOTIDE _PHOSPHODIESTERASE_ACTIVITY	0,002000675
	STEROID_BIOSYNTHETIC_PROCESS	0,001552398	CYCLIC_NUCLEOTIDE _PHOSPHODIESTERASE_ACTIVITY	0,002326236
	GROWTH_FACTOR_BINDING	0,002816334	ION_TRANSMEMBRANE _TRANSPORTER_ACTIVITY	0,002900393
	CELLULAR_LIPID_METABOLIC_PROCESS	0,003280416	TRANSMEMBRANE _TRANSPORTER_ACTIVITY	0,003027953
**KEGG pathway database interrogation**	MAPK_SIGNALING_PATHWAY	2,60E-02	INSULIN_SIGNALING_PATHWAY	4,83E-03
	CIRCADIAN_RHYTHM	0,030111343	ADHERENS_JUNCTION	0,006725233
	GLYCOSPHINGOLIPID BIOSYNTHESIS_GLOBOSERIES	0,035042657	TYPE_II_DIABETES_MELLITUS	0,020938416
	GLYCOSPHINGOLIPID BIOSYNTHESIS_GANGLIOSERIES	0,039949369	TASTE_TRANSDUCTION	0,022802817
	JAK_STAT_SIGNALING_PATHWAY	0,05392817	CALCIUM_SIGNALING_PATHWAY	0,054532557
