# Supplementary figures and images for: Molecular apocrine differentiation is a common feature of breast cancer in patients with germline PTEN mutations
Source: Breast Cancer Res. 2010 Aug 16;12(4):R63. doi: 10.1186/bcr2626 (PMC2949656; doi:10.1186/bcr2626)

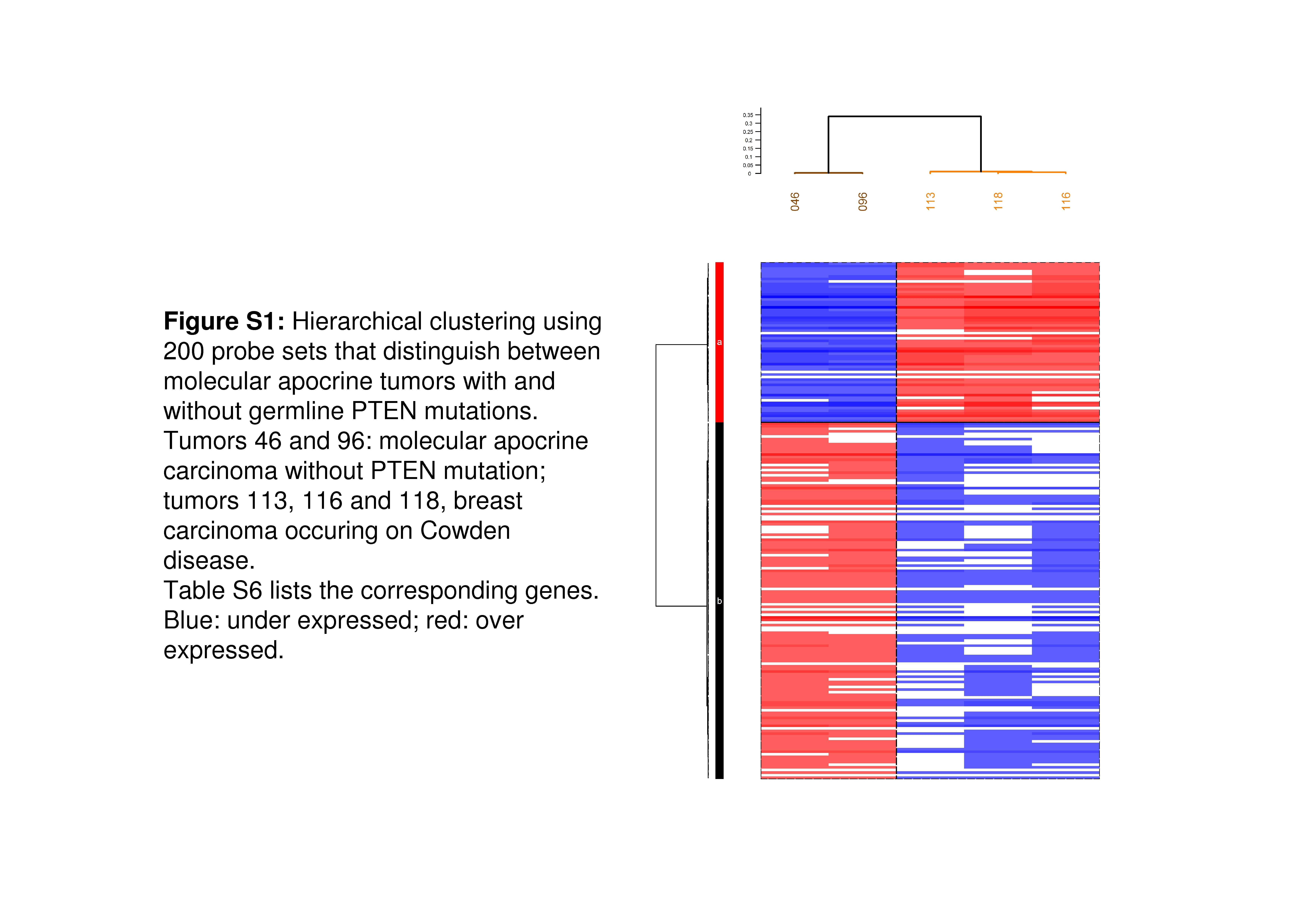

Supplement: Additional file 2 — Supplementary figures. Figure S1: Hierarchical clustering using 200 probe sets that distinguish Cowden from non Cowden apocrine breast cancers. [file bcr2626-S2.JPEG]
